# Transcriptomic landscape profiling of metformin‐treated healthy mice: Implication for potential hypertension risk when prophylactically used

**DOI:** 10.1111/jcmm.15472

**Published:** 2020-06-11

**Authors:** Yuhong Meng, Rui Xiang, Han Yan, Yiran Zhou, Yuntao Hu, Jichun Yang, Yuan Zhou, Qinghua Cui

**Affiliations:** ^1^ Department of Physiology and Pathophysiology Department of Biomedical Informatics Center for Non‐coding RNA Medicine MOE Key Lab of Cardiovascular Sciences School of Basic Medical Sciences Peking University Beijing China

**Keywords:** drug research, hypertension, metformin, systems biology, transcriptome

## Abstract

Recently, the first‐line anti‐diabetic drug metformin shows versatile protective effects against several diseases and is potentially prescribed to healthy individual for prophylactic use against ageing or other pathophysiological processes. However, for healthy individuals, it remains unclear what effects metformin treatment will induce on their bodies. A systematic profiling of the molecular landscape of metformin treatment is expected to provide crucial implications for this issue. Here, we delineated the first transcriptomic landscape induced by metformin in 10 tissues (aorta, brown adipose, brain, eye, heart, liver, kidney, skeletal muscle, stomach and testis) of healthy mice by using RNA‐sequencing technique. A comprehensive computational analysis was performed. The overrepresentation of cardiovascular disease‐related gene sets, positive correlation with hypertension‐related transcriptomic signatures and the associations of drugs with hypertensive side effect together indicate that although metformin does exert various beneficial effects, it would also increase the risk of hypertension in healthy mice. This prediction was experimentally validated by an independent animal experiments. Together, this study provided important resource necessary for investigating metformin's beneficial/deleterious effects on various healthy tissues, when it is potentially prescribed to healthy individual for prophylactic use.

## 1 INTRODUCTION

Metformin is the most popular first‐line drug for treating type 2 diabetes.^1^ As an effective hypoglycaemic agent, one of its main glucose‐lowering mechanism has been proposed to suppress hepatic glucose production via the activation of AMPK, a master regulator of both glucose and lipid metabolisms, and other pathways.^2^, ^3^ Besides, AMPK‐independent mechanisms have also been established, including but not limited to AMP‐dependent fructose‐1‐6‐bisphosphatase inhibition^4^ and redox‐dependent mitochondrial glycerol‐3‐phosphate dehydrogenase inhibition.^5^ Recently, metformin has been demonstrated to have versatile protection against many complex diseases beyond diabetes. For example, the hypoglycaemic and hypolipidemic effects of metformin also contribute to the risk reduction of cardiovascular diseases as evidenced by animal models.^6^ Clinical trial supports the protective effects of metformin against cardiovascular diseases like myocardial infarction in diabetic patients.^7^, ^8^ Moreover, metformin also inhibits several oncogenic pathways, and therefore, it has been suggested for the treatment of various cancers.^9^, ^10^, ^11^, ^12^ Indeed, novel usage of metformin against diseases like polycystic ovary syndrome,^13^ neurodegenerative diseases,^14^ lung fibrosis,^15^ multiple sclerosis ^16^ and fragile X syndrome^17^ have been continuously proposed, with novel mechanism‐of‐action proposed like exerting its anti‐cancer effect through mTOR regulation^18^ and anti‐inflammatory effect through IKKβ suppression,^19^ highlighting metformin as one of the top versatile drugs in the field.

Recently, metformin has been reported to have anti‐ageing effects in animal studies.^20^, ^21^, ^22^ A clinical trial which aimed at validating the anti‐ageing effects of metformin had also been newly launched.^23^ However, this novel proposal of metformin usage also signifies that the extensive investigations are needed to evaluate the impact of long‐term usage of metformin on healthy individual. Metformin is widely distributed to a variety of tissues after intake^24^ and therefore may elicit unexpected deleterious effects in these tissues. For example, the maternal exposure of metformin interferes the development of offspring's testis in normal mice.^25^ Indeed, concerns about the metformin's influence on offspring during polycystic ovary syndrome treatment has been aroused in clinical studies.^26^ Moreover, metformin's action is context‐dependent.^27^ For example, it has been reported that metformin exerts beneficial effects on breast cancer only in patients with metabolic syndrome but not in those without.^28^ In addition, the dual‐roles of AMPK signalling on cancer cells’ metabolism and growth have also been observed. Beyond inhibiting tumour cell growth, AMPK activation would also promote tumour cell growth by maintaining NADPH level under energy stress.^29^ Metformin has considerable influence on the composition of gut microbiota,^30^ and its therapeutic effect would be partly attributed to its effect on gastrointestinal tract and gut microbiota.^31^ However, the underlying mechanisms are complicated and involve gut‐oriented factors like FXR,^32^ and its impact on gut microbiota is clearly context‐dependent, varying among different treatment conditions or settings.^30^, ^32^, ^33^ Collectively, these findings suggested that although metformin has beneficial effects in many pathophysiological processes such as diabetes and cancers, it may also cause unexpected deleterious effects in physiological condition. Due to its versatile protective effects on many diseases and anti‐ageing potentials, metformin might be prescribed to healthy individual for prophylactic use or for lifespan prolonging purpose.^23^, ^34^ Long‐term clinical investigation with respect to metformin's diabetes prevention effect on high‐risk population like Diabetes Prevention Program Outcomes Study has been performed.^35^ One latest small‐scale clinical trial has stepped further to evaluate the potentials of metformin, together with other treatment, for anti‐ageing purpose on old population and observed positive effects on reversing aging‐related markers.^36^ However, another latest investigation has again raised the concern about the context‐dependent effect of metformin for anti‐ageing settings, where the metformin's effect is significantly modified due to the aerobic exercise training in older adults.^37^ Indeed, it remains largely unknown about the effects, particularly the unexpected deleterious effects of long‐term metformin treatment on healthy body and tissues. Which molecules will be influenced by metformin? How will these molecules change? What outcomes would be resulted from these changes? The answers to all these important questions are still unknown and thus should be emergently addressed.

To answer these questions, molecular landscape profiling, especially transcriptomic profiling, could be an efficient way. For example, calorie restriction is the most validated anti‐ageing factor.^21^ By comparing the mouse liver and skeletal muscle transcriptomes after metformin treatment, with those after calorie restriction, the similar anti‐ageing effect of metformin has been suggested and further experimentally validated.^21^ However, current available transcriptome data about metformin are performed under pathophysiological conditions such as obesity and diabetes. Furthermore, these transcriptome data often cover very few tissues. To date, no systematic transcriptome profiling of the normal tissues after metformin treatment is currently available, which limits the evaluation of the potential beneficial and deleterious effects of metformin treatment on healthy tissues. In the current study, normal healthy mice had been orally treated with moderate dose of metformin for one month, and then the transcription profiles in 10 metformin‐treated tissues had been determined using high throughout RNA sequencing. Computational models had been further developed to predict both the beneficial and deleterious effects of metformin in 10 tissues based on the transcription profiles, highlighting the risk for inducing unexpected diseases in normal mice. The predictions for metformin‐induced hypertension and cardiac hypertrophy and in healthy mice were further validated by independent animal experiments. Overall, the transcriptome data delineated a landscape of metformin‐induced molecular profiles in healthy condition and the findings provided a useful resource for interrogating the potential effects of long‐term metformin usage in healthy human beings.

## 2 MATERIAL AND METHODS

### 2.1 Experimental animal details

Eight‐week‐old male/female C57BL/6 and old male mice (60‐62 weeks) were fed on normal diet under the condition of constant temperature of 25°C. The mice were randomly divided into the experimental and control groups. Metformin hydrochloride tablets (Glucophage, 0.5 g/tablet, manufactured by Sino‐American Shanghai Squibb Pharmaceuticals Ltd.) were dissolved in double‐distilled water to the concentration of 30 mg/mL. Generally, when metformin was used to treat animals, the daily dosage varied from 100 to 600 mg/kg bodyweight.^38^, ^39^, ^40^ In the current study, the experimental group of mice were orally administrated by metformin at the daily dosage of 300 mg/kg bodyweight for one or three months, whereas the control group of mice were orally administrated with the same volume of double distilled water. According to the previous researches, it has been suggested that the effect of the standard dose of metformin used in humans for the treatment of type‐2 diabetes (~20 mg/kg) is equivalent to that of the ~250 mg/kg dose used in mice, despite the absolute dose in mice is considerably higher than the dose in humans.^41^

### 2.2 High‐throughput transcriptome profiling

After 30 days of treatment, the mice were anesthetized via pentobarbital injection, killed and the tissues were harvested, flash frozen on dry ice. Total RNA was extracted by using Qiagen RNeasy kit, prepared with Illumina TruSeq Stranded Total RNA Library Prep kit under the manufacturer's guideline. RNA libraries were prepared for sequencing using standard Illumina protocols and sequenced by Illumina HiSeq 2500 platform (provided by BerryGenomics).

Raw reads were trimmed for adaptor sequence and masked for low‐complexity or low‐quality sequence, including those containing more than 10% of unknown nucleotides or more than 50% of low‐quality (ie Q‐value no larger than 20%) bases and then were filtered against rRNA database to remove potential rRNA contamination. Reads that passed the above filtration and quality control were mapped to GRCm38 genome (Ensembl release 84) by TopHat2 software (https://ccb.jhu.edu/software/tophat, version 2.1.1) with custom parameters “‐g 1 ‐r 50 ‐‐mate‐std‐dev 80 ‐‐no‐coverage‐search ‐‐phred64‐quals ‐‐keep‐fasta‐order”. Gene abundances were quantified by RSEM software (http://deweylab.github.io/RSEM, version 1.2.19) with the recommended command line parameters in the published protocol. The gene expression level was normalized by using the fragments per kilobase of transcript per million mapped reads (FPKM) method, where the number of fragments mapped to the specific gene was normalized by the total number of fragments that mapped to reference genes and the number of bases on this gene.

### 2.3 Transcriptome profile comparisons

The transcriptome profiles (FPKM normalized expression values) were clustered by hierarchical clustering method and principle component analysis (PCA) by using the hclust and pca functions of R (https://www.r‐project.org, version 3.4.0) and were illustrated by using the pheatmap and ggplot2 packages of R, respectively. For comparative analysis with public transcriptome data, we defined the signature of transcriptome alteration by one treatment as the array of log_2_(fold change) comparing experimental group vs control group. The transcriptomic signatures were comprehensively deduced from the curated transcriptome data in GEO database (queried from September, 2016 to December, 2017). The samples in one transcriptome data set were manually assigned to experimental or control groups based on the annotations on GEO data sets and samples, and the related publication if applicable. For two‐colour arrays with paired case‐vs‐control design, the fold changes were directly deduced from the signal ratio. Two‐colour arrays with universal reference were treated as the same as one‐colour array. The fold changes from one colour arrays and RNA‐seq data were deduced from the normalized expression values. All expression values, if not log_2_‐transformed, were transformed before calculating fold changes. Finally, the fold change signatures by metformin in this study and the curated signatures were compared by Spearman's correlation coefficient (SCC), in which only genes shared by two studies were taken in consideration. We removed the redundant signatures (inter‐signature SCC > 0.5 and of the same topic) and corrected the *P*‐value by the Bonferroni method for multiple hypothesis correction.

### 2.4 Identification and analysis of DEGs

The differentially expressed genes (DEGs) were identified by edgeR package of R with default parameters. Because the transcriptome profiles were used for hypothesis generation rather than validation, a relaxed threshold of *P* < .05 was applied. For functional enrichment analysis, mouse DEGs were firstly mapped to human orthologous genes based on the mapping function provided by g:Profiler (http://biit.cs.ut.ee/gprofiler). The functional enrichment analysis for GAD disease gene sets and KEGG pathways was performed by using the DAVID tool (https://david.ncifcrf.gov, version 6.8) with human official gene symbols as the input. We also compared the DEGs with curated gene sets in response to chemical and genetic perturbations from the MSigDB online platform (http://software.broadinstitute.org/gsea/msigdb). Finally, the overrepresented transcription factors were analysed by using the enrichment analysis tool in ChIP‐Atlas platform (https://chip‐atlas.org). The result is visualized by using the gplots and riverplot packages in R.

The human orthologous genes of mouse DEGs were further mapped onto the human signalling network. The latest version of the human signalling network was obtained from the website of Wang lab (http://www.cancer‐systemsbiology.org/, update v7). The network topology metrics like PageRank centrality and betweenness centrality was calculated by the igraph package in R. Furthermore, the drug target genes from the DrugBank database (https://www.drugbank.ca) and the DSigDB database (http://tanlab.ucdenver.edu/DSigDB/DSigDBv1.0) were also mapped onto the network. Then, the average distance between one DEG set *D_i_* = 1*, …, m* and one drug target set *T_j_ *= 1, …, *n* was assigned by using the formula below:

$$\text{dist}\left(D,T\right)=\sum_{i=1}^{m}\min_{T_{j}\in T}\left[\text{dist}\left(D_{i},T\right)\right]/m$$

To assess the significance of the network distance, equal number of false drug targets were randomly selected from the network for 1000 times and the false discovery rate (FDR) was assessed accordingly. For each tissue and each drug, such analysis was repeated six times with the different input combination of DEG sets (up‐regulated genes only, down‐regulated gene only or both of them) and drug target sets (DrugBank or DSigDB). The results supported by at least one of the DEG sets and by both of the drug target sets were retained. The properties and side effect of the drugs were analysed by DrugPattern tool (http://www.cuilab.cn/drugpattern/). We also calculated the SCCs between metformin's transcriptomic signatures from this study and the drug‐induced transcriptomic alteration signatures from the LINCS CMap project (https://clue.io/lincs). Only signatures from the same tissue origin were compared, and the *P*‐values were corrected by Bonferroni method.

### 2.5 Measurement of blood pressure

Blood pressure and heart rate of mice were measured by HD‐X10 implantable transmitter after 3‐month metformin treatment. Mice after 3‐month metformin treatment were anesthetized with 1% sodium pentobarbital (i.p. injection). The left carotid artery was isolated from the surrounding tissue. The flank of the mouse was bluntly isolated with a vascular clamp to form a subcutaneous pocket. Rinse the pocket with sterile saline and place the HD‐X10 transmitter in it. The skin incision was sutured. Blood pressure levels were measured after 5 days. When blood pressure levels were measured by tail‐cuff method, mice after 3‐month metformin treatment were fixed in the canister in 37°C. Blood pressure and heart rate of mice were measured when mice were stabilized. Blood pressure and heart rate of every mouse were averaged from 10 measurements.

## 3 RESULTS

### 3.1 Transcriptomic landscapes of 10 healthy tissues induced by one‐month moderate metformin treatment

**FIGURE 1:**
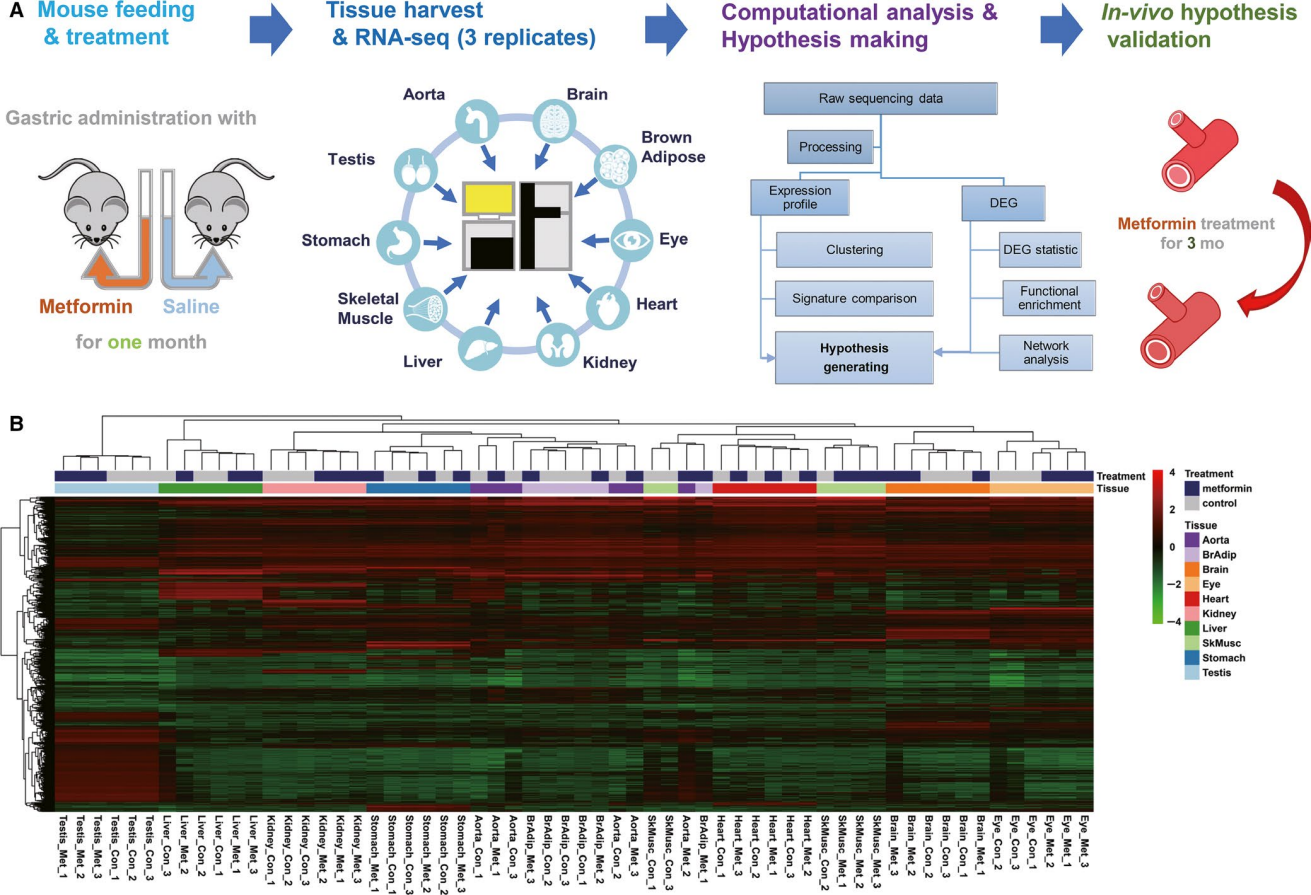
The overview of the transcriptome profiles after metformin treatment across ten healthy tissues. A, Workflow of this study. Normal mice were randomly split into two groups, the experimental (one‐month treatment of metformin) and control groups. The transcriptome across 10 tissues was profiled, and subsequent integrative computational analyses (clustering, signature comparison, functional association and network‐based analysis with drug target association) were performed to generate hypothesis about the potential beneficial and adverse effect of metformin treatment on normal mice. Finally, the hypothesis about cardiovascular side effects was validated in vivo with prolonged period (3 mo) of metformin treatment. B, Heat map illustrating the clustering of transcriptome profiles of differentially expressed genes, with or without metformin treatment, across 10 tissues

**FIGURE 2:**
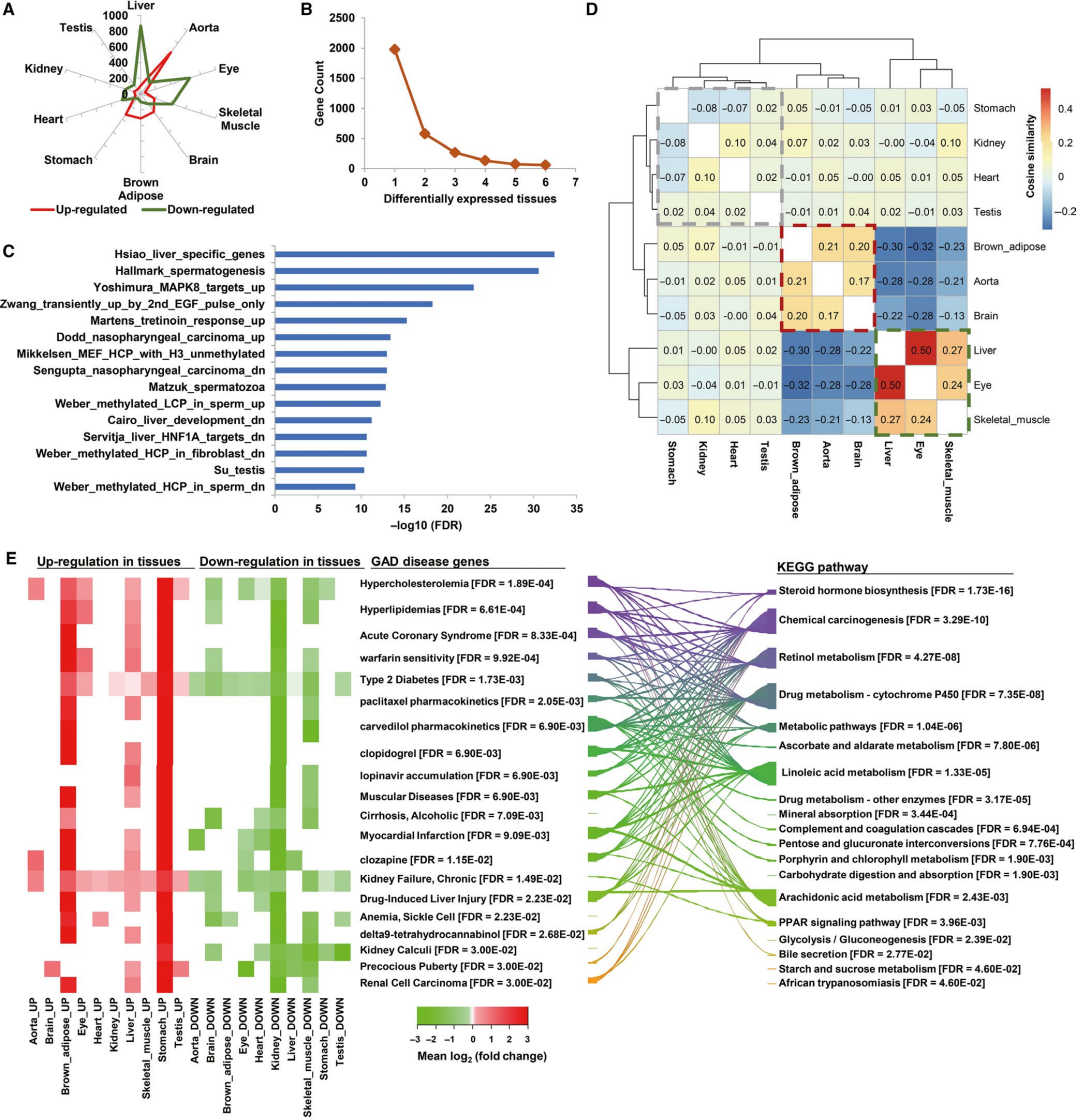
The functional associations of differentially expressed genes (DEGs). A, Counts of DEGs across ten tissues. Tissues are sorted according to their total DEG number. B, Distribution of DEGs shared by multiple tissues. C, Top 15 gene sets (MSigDB hallmark and chemical/genetic perturbation gene sets) showing significant overlap with the frequently shared DEGs between tissues. D, The clustering of tissues based on the cosine similarity (shown in each box) between their DEGs. E, The GAD disease gene and KEGG pathway enrichment of the frequently shared DEGs. The redundant GAD disease gene sets are not shown. The width of the strap between GAD and KEGG terms correlates with the number of shared genes between them. Only straps indicating significant overlaps (Fisher's exact test, *P* < .05) are shown

To investigate the potential impact of metformin on various healthy tissues, normal mice with chow diet were treated with moderate dosage of metformin (300 mg/kg) for one month. Ten tissues (aorta, brown adipose, brain, eye, heart, liver, kidney, skeletal muscle, stomach and testis) were harvested, and the transcriptomes were profiled by high‐throughput RNA‐seq (Figure 1A). Each treatment (metformin or saline control) plus tissue combination were fortified by three biological replicates, which results in 60 transcriptome profiles depicting the landscape of transcriptome alteration by metformin treatment. Because the transcriptome profiles were used for hypothesis generation rather than validation, a relaxed threshold of *P* < .05 was applied. In all, there are 3079 genes showing differential expression in at least one of the 10 tissues (that is to say, there are 3079 genes in total which show differential expression in at least one of the 10 tissues). The overall similarity between transcriptome profiles of these genes is summarized in the heat map presented in Figure 1B. The transcriptome profiles are clustered primarily according to their tissue origin, indicating the transcriptome alteration by metformin are tissue‐dependent. This observation can be confirmed by principle component analysis (PCA), where the samples are largely aggregated according to their tissue origin (Figure S1). The extent and overall direction of the transcriptome alteration induced by one‐month metformin treatment varies among tissues, in which liver, eye and skeletal muscle exhibit the highest numbers (867, 652 and 418, respectively) of down‐regulated genes, while aorta, stomach, brown adipose and brain show the highest number (651, 323, 312 and 280, respectively) of up‐regulated genes (Figure 2A).

### 3.2 Functional and disease associations of differentially expressed genes

In line with the transcriptomic profile clustering results, only few DEGs (261 genes) are shared by more than three tissues (Figure 2B), indicating the tissue specificity of the transcriptomic responses to metformin treatment. To probe the functional and disease associations of these shared DEGs, they were firstly compared with the curated gene sets about chemical and genetic perturbations from the MSigDB database. The top significant overlapped genes sets are listed in Figure 2C. Liver is one of the primary target organs of metformin and harbours a considerable fraction of the DEGs in our study. Accordingly, the liver selective genes and genes down‐regulated in early liver development are overrepresented in the shared DEGs. Besides, genes down‐regulated in the diethylnitrosamine treatment‐induced or *E2F1* overexpression‐induced hepatocellular carcinoma models and *HNF1A* knockout model of type 1 diabetes are also overrepresented, supporting the liver protective roles of metformin.^42^, ^43^, ^44^ Moreover, genes that are de‐regulated in nasopharyngeal carcinoma are overrepresented, in line with the recently discovered anti‐nasopharyngeal carcinoma action of metformin.^45^ Finally, gene sets related to spermatogenesis are also presented in the top list. The metformin's impact on male reproduction has gained increasing notice recently,^46^ and our data suggest that the risk of male fertility reduction after metformin treatment deserves serious assessments, both clinically and on animal models.

We further clustered the tissues based on their correlation of DEGs (Figure 2D). The aorta, brain and brown adipose, in which genes are largely up‐regulated by metformin treatment, are grouped as one module (Mod_up). Similarly, the tissues where genes are largely down‐regulated, including eye, liver and skeletal muscle, are grouped as another module (Mod_down). Finally, the rest tissues (stomach, heart, kidney and testis) showing the DEG pattern in‐between form a loosely connected module (Mod_inbetween). To be more precise, the shared DEGs and the DEGs exclusively presented in either of three modules are analysed by the DAVID tool respectively, and the top significant associations are depicted in Figure 2E and Figure S2, respectively. We also analysed the overrepresented transcription factor behind each DEG module, and the results are shown in Figure S3A‐C. A detailed discussion of the results is available in Appendix S1, where the significant associations with cardiovascular diseases like hypertension are highlighted and will be experimentally validated in the last section.

### 3.3 Comparative analysis of the transcriptomic signatures predicts potential protective and deleterious effects of metformin on 10 healthy tissues

To investigate whether the metformin‐induced transcriptome changes could imply beneficial or deleterious effects, for each tissue, the metformin‐induced gene expression alteration signature (metformin signature for short) is calculated as the fold changes of gene expression comparing metformin‐treated samples vs control samples. The metformin signatures are then compared with our curated gene signatures of various physiological or pathophysiological conditions from the GEO database. The curated signatures have covered some typical transcriptomic changes related to disease model, drug treatment and lifestyle intervention. The signatures significantly correlated with the metformin signatures are listed in Table S1. In the next section, we will focus on the correlated signatures in liver and heart. The predicted potential protective and deleterious effects of metformin on the other eight tissues are described in Appendix S1, Figures S4 and S5.

### 3.4 Potential protective and deleterious effects of metformin on healthy liver and heart

**FIGURE 3:**
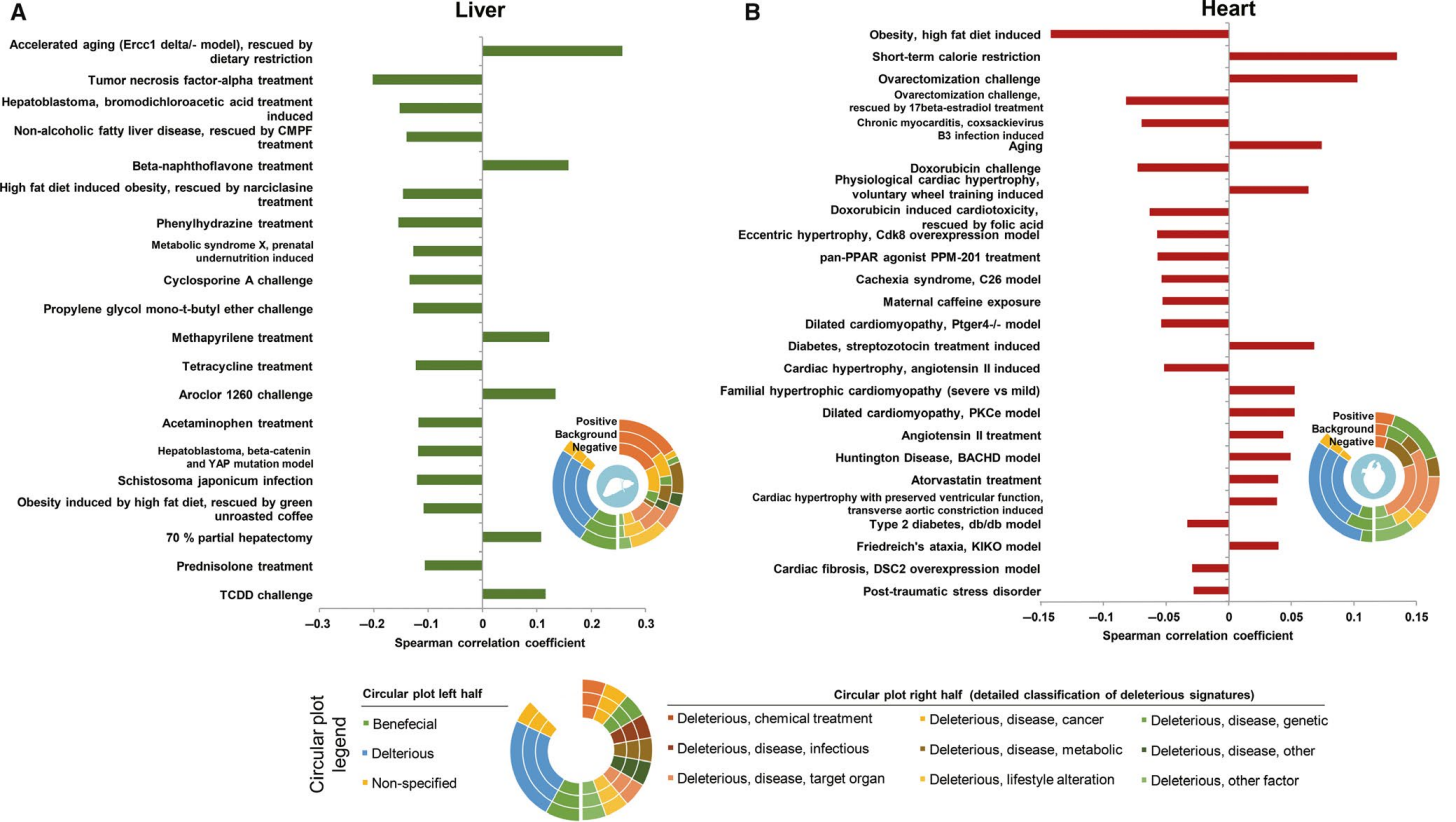
The significant correlations between metformin's transcriptomic signature and curated reference transcriptomic signatures in liver and heart. A, Correlations of transcriptomic signature (gene expression fold changes) between metformin‐treated normal mice and other treatments or disease models in liver, the top 20 significant correlations are shown. B, The significant correlations in heart

Since the liver is one primary known target tissue of metformin, we first performed the comparison in liver (Figure 3A). Generally, the metformin signature is negatively correlated with multiple disease models like bromodichloroacetic acid treatment induced hepatoblastoma (SCC = −0.151, FDR = 1.32E‐78), prenatal undernutrition induced metabolic syndrome X (SCC = −0.127, FDR = 9.57E‐57) and *Schistosoma japonicum* infection (SCC = −0.127, FDR = 1.41E‐40) and liver toxic or carcinogenic drug treatments like phenylhydrazine, propylene glycol mono‐t‐butyl ether, tetracycline and acetaminophen (SCC < −0.1, FDR < 1.0E‐40), which are in line with the literature‐reported hepatoprotective roles of metformin.^42^, ^43^, ^44^ However, there is no prominent overall tendency for negative correlation between metformin signature and deleterious signatures (Figure 3A). Indeed, positive correlations with liver toxic or carcinogenic drug treatments like Aroclor 1260 (SCC = 0.134, FDR = 8.98E‐47) and TCDD (SCC = 0.116, FDR = 9.04E‐35) are also observed. This observation is noteworthy, as previous comparison using high‐fat diet (HFD) treated mice model has indicated highly consistent beneficial correlations of the metformin intervention signature.^47^ Therefore, the impact of metformin on mice liver seems context‐dependent, and the influence of long‐term usage of metformin on healthy livers would be assessed to rule out its potential liver toxicity. Besides, β‐naphthoflavone is a known immune inhibitor that exerts anti‐inflammatory effect by suppressing TNF‐α pathway,^48^ and metformin signature positively correlates with β‐naphthoflavone treatment signature (SCC = 0.158, FDR = 4.49E‐65), but negatively correlates with TNF‐α treatment signature (SCC = −0.201, FDR = 2.45E‐106), supporting its anti‐inflammatory roles in liver.^49^ Finally, we note the complicated relationship between metformin signature and ageing signature. On the one hand, metformin signature exhibits obvious similarity with dietary restriction anti‐ageing intervention (SCC = 0.258, FDR = 2.72E‐231), mimicking the previous investigation.^21^ On the other hand, it also positively correlates with the ageing signature (old mice vs young mice, SCC = 0.093, FDR = 2.02E‐29). This is not likely coincidental as positive correlations with ageing signature can also be observed when using ageing signatures from the other studies (Table S1). Indeed, although metformin has been proposed as an anti‐ageing drug,^23^ its effect on healthy lifespan is complicated by the onset and dosage of treatment.^50^ It is possible that the context of metformin treatment in this study is not appropriate for anti‐ageing assay, for example one study using the same dosage of metformin failed to expand healthy lifespan of mice.^51^ We also analysed the overrepresented transcription factor behind DEGs in liver, and the results suggest that transcription factors like TAF1 (FDR = 1.99E‐41), BRD2 (FDR = 1.80E‐39) and NELFA (FDR = 1.44E‐36) are likely involved in the gene expression regulation in response to the metformin treatment (Figure S3D).

Metformin has been implicated as a protective reagent against cardiovascular diseases in diabetic context.^8^, ^52^ Indeed, metformin signature also negatively correlates with the doxorubicin induced cardiotoxicity (SCC = −0.073, FDR = 4.83E‐15) and Ptger4‐/‐ model of dilated cardiomyopathy (SCC = −0.054, FDR = 4.19E‐8). Nevertheless, as indicated by the circular plot, the fraction of deleterious signatures among the positive correlated signatures are intuitively higher than the background (Figure 3B). For instance, noticeable positive correlations with the cardiac hypertrophy signatures, induced either by voluntary wheel training (SCC = 0.064, FDR = 3.70E‐14), by familial genetic background (SCC = 0.053, FDR = 5.59E‐07), or by transverse aortic constriction (SCC = 0.039, FDR = 5.89E‐5), appear in the top list of correlated signatures. Interestingly, metformin signature also positively correlates with the ovarectomization challenge signature (SCC = 0.103, FDR = 4.50E‐36). Oestrogen signalling has been implicated as one of the key protective pathways against cardiac hypertrophy.^53^ One plausible hypothesis is that metformin would perturbate oestrogen signalling or its downstream effectors and therefore induce cardiac hypertrophy in normal mice. We also analysed the overrepresented transcription factor behind DEGs in heart, and the results suggest that transcription factors like NELFE (FDR = 3.97E‐43), NELFA (FDR = 2.50E‐42) and SMAD3 (FDR = 5.93E‐40) are likely involved in the gene expression regulation in response to the metformin treatment (Figure S3E).

### 3.5 Network analysis of differentially expressed genes indicates potential effects of metformin on blood pressure

**FIGURE 4:**
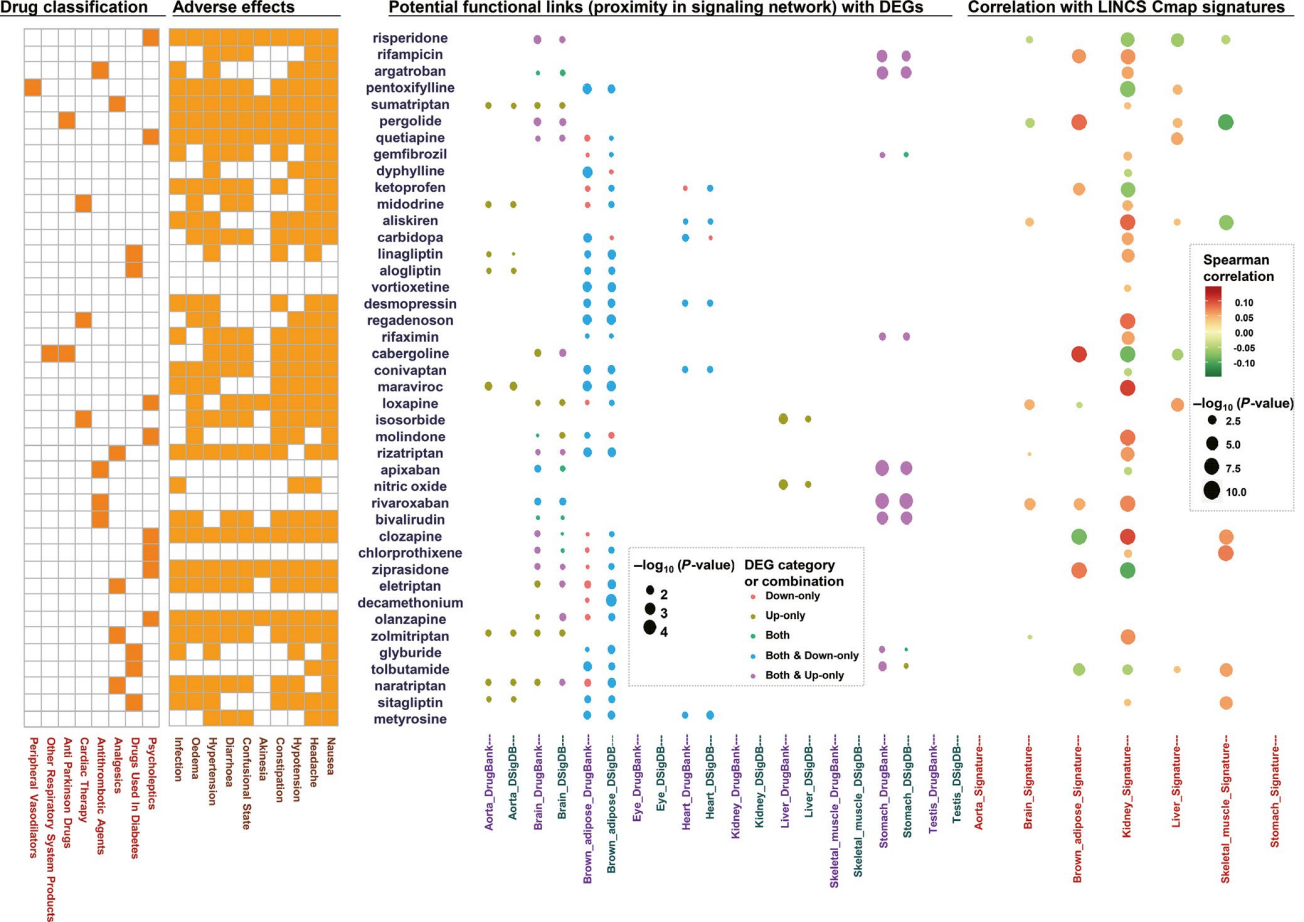
Potential links between drugs and DEGs in context of human signalling network. If the distance from one drug's known physical target genes to the DEGs from one specific tissue is significantly closer than random expectation, a potential functional link between the drug and the DEGs are assumed. The top five functionally linked drugs from the analysis in each tissue, together with those shared by multiple tissues, are shown in the bubble plot at the second panel to the right. The Spearman's correlation between the metformin's transcriptomic signature (differential expression induced by metformin) and those of other drug perturbations in the same tissue (differential expression induced by other drugs in cell lines representing the same tissue) from LINCS CMap data set are shown as the bubble plot at the first panel to the right. The drug classification and adverse effects are indicated by the heat maps at the left panels

We further investigate the functional associations of the DEGs induced by metformin in the human signalling network context. The DEGs induced by metformin treatment across 10 tissues were mapped onto the human signalling network. Analysis of canonical network topology metrics indicates that the DEGs have higher PageRank centrality and betweenness centrality (Figure S6A‐B), indicating that the DEGs tend to settle in the nexuses of signalling pathways. Nevertheless, such analysis does not specify the functional associations of DEGs. Therefore, we further mapped the curated drug targets from DrugBank and DSigDB databases to the human signalling network and compared the distance between DEGs and known drug targets with the random expectation (see Methods for details). If the DEGs are closer to the *bona fide* target genes of one drug, in comparison with the randomized false targets, a potential functional linkage between this drug and metformin is suggested. Such analysis is performed three times for each set of drug targets, using the up‐regulated genes, down‐regulated genes and both of them as the input, respectively. Figure 4 reports the top consensus results. Because discrepancy in the drug target annotations, the detailed results may not be identical for the two databases. This top consensus list of genes would imply some interesting hypothesis. First, it is enriched (Fisher exact test, *P* = 1.47E‐5) for the drugs that have known drug‐drug interactions with metformin, including clozapine, olanzapine, quetiapine, risperidone, ziprasidone, glyburide and tolbutamide (Figure S6C). Considering the limitation of the curated drug‐drug interactions, we also employed the predicted drug‐drug interactions from DeepDDI project.^54^ Among the top drug list, metformin is predicted to have drug‐drug interactions with a considerable fraction of these top drugs (18 out of 42, Fisher's exact test, *P* = 1.92E‐10; Table S2). Second, the brown adipose stands out as the tissue in which the highest number of potential functional linkage between drugs are observed. We used DrugPattern tool to analyse the shared properties of these drugs. Among these drugs, anti‐diabetic drugs are overrepresented (Figure 4; FDR = 6.23E‐7). Third, links with psycholeptic drugs are enriched in both brown adipose and brain (FDR = 1.02E‐7 and 1.78E‐10, respectively). Indeed, clinical investigations have indicated the effectiveness of metformin against the side effects of psycholeptic drugs.^55^ Our data would provide clues for interpreting the mechanism of actions behind such effect.

We further focused on the potential deleterious effects of metformin treatment on healthy mice. Indeed, links with drugs that have nausea side effect, one of the most prominent known side effects of metformin, are enriched in stomach (FDR = 1.22E‐3). Moreover, the drugs showing hypertension side effect are enriched in brown adipose (FDR = 1.22E‐3) and more importantly in heart (FDR = 1.11E‐3). The functional link with four drugs (desmopressin, ketoprofen, aliskiren and conivaptan) that have such side effects are highlighted (Figure S6D). To be more precise, we further compare the previously mentioned metformin's transcriptomic signatures (Table S1) with the drug‐induced transcriptomic signatures from LINCS CMap data set. Metformin signatures turn out to be significantly correlated with most of the top drugs’ signatures in at least one tissue (Figure 4). Intuitively, a positive correlation would imply similar therapeutic and/or side effect between drugs. Indeed, metformin signature is prone to show positive correlations with the signatures of the above‐mentioned four metformin‐linked drugs with hypertensive side effect (Figure 4), implying that metformin treatment on healthy mice may arouse side effect similar to these drugs. Another interesting example is clozapine. Potential functional links with this drug are observed in brain and brown adipose, while significant positive correlations of transcriptomic signatures can be found in kidney and skeletal muscle (Figure 4). A clinical study indicates that clozapine has noteworthy effect for elevating blood pressure.^56^ Interestingly, according to the DeepDDI’s prediction, metformin and clozapine, when used together, may increase the risk of adverse effects (Table S2). Given the high risk for a variety of fatal diseases of hypertension and the increasing medication rate of metformin, it is emergently needed to further experimentally investigate the long‐term usage of metformin of healthy mice's blood pressure.

### 3.6 Experimental validation of the prediction that long‐term metformin treatment would induce hypertension in healthy mice

**FIGURE 5:**
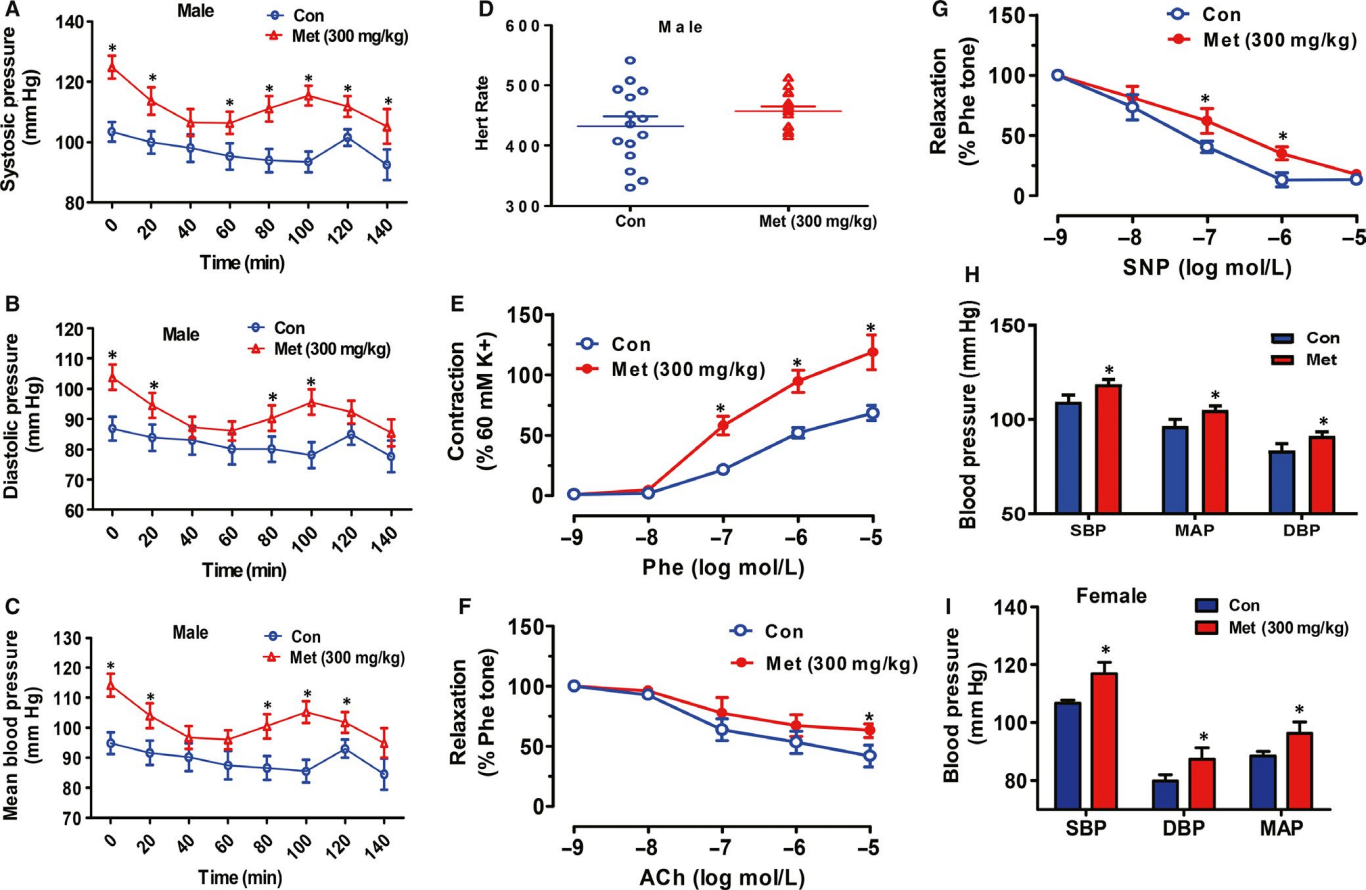
Metformin treatment increased vascular constriction and blood pressure levels in young mice. (A‐C) Metformin treatment increased: systolic blood pressure (A), diastolic blood pressure (B) and mean artery pressure (C) in young male mice. (D) Metformin treatment had little effect on heart rate of young male mice. 8‐ to 10‐week‐old male mice were daily treated with metformin (300 mg/kg bodyweight) or water for 3 mo. Blood pressure levels were measured by HD‐X10 implantable transmitter (Data Sciences International, DSI). Con, control mice treated with water; Met, mice treated with metformin. N = 12‐15, *P < .05 vs control mice treated with water. (E) Thoracic arteries of metformin‐treated young male mice had stronger constriction in response to phenylephrine than those of control mice. (F‐G) Thoracic arteries of metformin‐treated young male mice had weaker: endothelial‐dependent (F) and endothelial‐independent relaxation (G) with those of control mice. Phe, phenylephrine; SNP, sodium nitroprusside dehydrate; Ach, acetylcholine. N = 5, *P < .05 vs control mouse arteries. (H‐I) Metformin treatment increased blood pressure levels in young: male (H) and female (I) mice as measured by tail‐cuff method. N = 8‐10, *P < .05 vs control mice treated with water. 8‐ to 10‐week‐old male or female mice were daily treated with metformin (300 mg/kg bodyweight) or water for 3 mo. The blood pressure levels were measured by tail‐cuff method. DBP, diastolic blood pressure; MAP, mean artery pressure; SBP, systolic blood pressure. N = 8‐10. The results are presented as the mean ± SEM. Statistical significance of differences between groups was analysed by *t* test. *P < .05 vs control mouse arteries

The above bioinformatics analyses of transcriptome alteration in mouse healthy tissues treated with metformin for 1 month revealed that long‐term administration of metformin tended to cause a number of diseases including hypertension. To validate the prediction that long‐time use of metformin causes hypertension, mice were orally treated with metformin for 3 months, and then the relaxation and constriction, and blood pressure levels were measured. In young male normal C57BL/6 mice, 3‐month administration of metformin decreased bodyweight and fasting blood glucose levels (Figure S7A‐B). However, metformin treatment had no significant effect on overall glucose tolerance (Figure S7C‐D). In young male mice, 3‐month metformin treatment significantly increased systolic, diastolic and mean blood pressure as measured by HD‐X10 implantable transmitter (Figure 5A‐C). Metformin treatment had little effect on the heart rate of young male mice (Figure 5D). To further validate these observations, the effects of metformin treatment on the relaxation and constriction of mouse arteries were measured. Consistently, the results indicated that after 3‐month metformin treatment, the constriction of mouse thoracic arteries in response to phenylephrine was significantly increased when compared with control mouse arteries (Figure 5E). In contrast, the relaxation of mouse thoracic arteries in response to *sodium* nitroprusside dehydrate (SNP) and acetylcholine (Ach) was significantly impaired (Figure 5F‐G). These findings supported the blood pressure level increases after metformin treatment on young male mice. In support, the tail‐cuff method also revealed that 3‐month metformin treatment significantly increased blood pressure levels in young male mice (Figure 5H). Consistently, 3‐month metformin treatment similarly increased blood pressure levels in young female mice (Figure 5I). These findings suggested that long‐term metformin administration did not impair renal functions in young mice.

**FIGURE 6:**
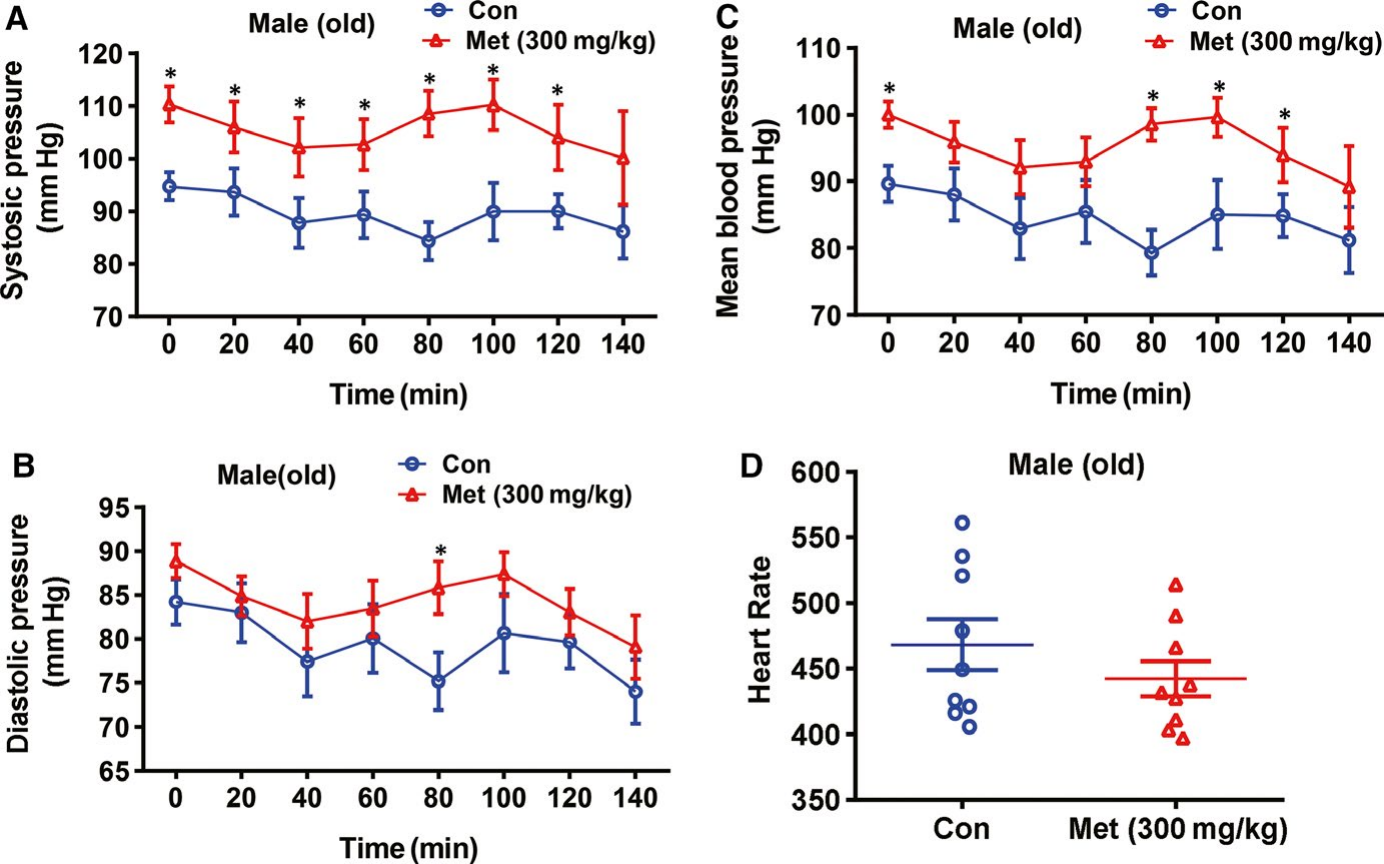
Metformin treatment increased blood pressure levels in old mice. (A‐C) Metformin treatment increased: systolic blood pressure (A), diastolic blood pressure (B) and mean artery pressure (C) in old male mice. (D) Metformin treatment had little effect on heart rate of old male mice. 60‐ to 62‐wk‐old male mice were daily treated with metformin (300 mg/kg bodyweight) or water for 3 mo. Blood pressure levels were measured by HD‐X10 implantable transmitter. Con, control mice treated with water; Met, mice treated with metformin. N = 9. The results are presented as the mean ± SEM. Statistical significance of differences between groups was analysed by *t* test. *P < .05 vs control mice treated with water

To further verify the metformin‐induced hypertension in normal mice, we investigated the effects of metformin treatment on old male mice as well. As a result, 3‐month administration of metformin had little effects on bodyweight and white adipose weight/bodyweight ratio (Figure S8A‐B). Moreover, metformin treatment also had limited effect on fasting blood glucose level and overall glucose tolerance (Figure S8C‐D). In old male mice, 3‐month metformin treatment significantly increased systolic, diastolic and mean blood pressure as measured by HD‐X10 implantable transmitter (Figure 6A‐C). Metformin treatment had little effect the heart rate of old young mice (Figure 6D). Given that healthy people may take a less dosage of metformin treatment, it is also important to investigate whether less dose of metformin treatment also can induce hypertension. Indeed, we showed that 100 mg/kg dose of metformin treatment also induces hypertension in healthy young and old mice (Figure S9). Overall, these findings further support that long‐term use of metformin can cause hypertension in normal mice.

## 4 DISCUSSION

In the past decades, metformin had been reported to exert beneficial effects on various diseases beyond diabetes. More recently, metformin has also been shown to have anti‐ageing effects and prolong lifespan.^20^, ^21^, ^22^ Metformin is potentially prescribed to healthy individual for delaying ageing and prolonging lifespan based on recent studies,^57^, ^58^ and some healthy individuals start to take metformin as prophylactical use or anti‐aging. However, the effects of metformin treatment on healthy tissues remain largely unknown. Therefore, it is of great significance and necessarily to evaluate the impact of long‐term administration of metformin on various tissues under physiological condition. In the current study, we depicted the impact of long‐term treatment with metformin on the transcriptomic landscape covering 10 tissues of normal mice using high‐throughput sequencing. Bioinformatics analyses including gene set association analysis, comparative transcriptomic signature analysis and network analysis of drug targets predicted that long‐term administration of metformin exerts both beneficial and deleterious effects on various tissues. For example, metformin likely exerts beneficial effects on liver by counteracting the gene expression alterations induced by carcinogenic drugs or infectious agents. In brain, metformin could partly reverse the transcriptomic signatures of multiple diseases including Parkinson disease, Alzheimer's disease and autism. This beneficial potential is also supported by the predicted functional links between metformin and several psycholeptic drugs. Metformin seems to exhibit the most prominent beneficial effect on adipose, by mimicking the transcriptomic changes during white‐to‐brown adipose transition and caloric restriction. In fact, metformin also shows several potential functional links with anti‐diabetic drugs in adipose tissue.

Though our analysis has suggested various potential beneficial roles of metformin, on the other hand, however, metformin also has the potentials to result in several diseases including hypertension in normal mice. One noticeable result highlights the hypertension risk, which has been implied by the overrepresentation of cardiovascular disease‐related gene sets, positive correlation with hypertension‐related transcriptomic signatures and the associations of drugs with hypertensive side effect together. Independent experimental validation further confirmed the bioinformatics prediction that long‐term metformin treatment increased blood pressure in normal mice. Although in diabetic patients, metformin administration could improve left ventricular diastolic function^59^ and reduce blood pressure levels,^60^ which might be due to the improvements of insulin resistance, hyperglycaemia, hyperlipidemia and endothelial dysfunction. However, metformin's anti‐hypertension effect among non‐diabetic populations is still under debate.^61^, ^62^ At present, there is no direct evidence on blood pressure changes in the normal population after taking metformin. Metformin is the drug of choice for type 2 diabetes. The dosage of metformin we used here is 300 mg/kg. The dose of ~300 mg/kg is a popular mouse dosage, which is relatively comparable with the human dose of 20 mg/kg. Given that hypertension is a major risk factor for a variety of fatal diseases including cardiovascular disease and stroke, the risk of long‐term usage of metformin to elevate blood pressure should be especially noted when prophylactically used.

Nevertheless, there are several technical limitations in current transcriptome analysis. First, in order to find the potential beneficial and deleterious effects of metformin in more healthy tissues, we only assigned three biological replicates for each sample group. As the result, a few sample group still exhibited notable intra‐group variance (Figure 1B). Though here we cannot remove the potential outlier because there are not enough replicates per group, more replicates are required for further detailed investigation of metformin's effect and mechanisms on one particular healthy tissue. Second, even 10 tissues were explored, there are still some notable tissues that deserve investigations. For example, the emerging effect of metformin on gut and gut microbiome would be explored in the future. Finally, the cell types influenced by metformin are not clear as shown in the bulk RNA‐seq transcriptome. There are several successful applications of bulk RNA‐seq transcriptome deconvolution method to identify the altered cell types (eg to identify immune cell infiltration in tumours).^63^ We have also tried to deconvolute our transcriptome profile by using xCell method.^64^ However, we could only find few cell types exhibiting significantly altered deconvolution scores and there is no agreement between different tissues (Table S3). Indeed, few interesting hypothesis could be noted, for example we noted the increased muscle cell and decreased adipocyte cell in skeletal muscle tissue after metformin treatment, which is in line with known effect of metformin.^50^ Nevertheless, the cell type‐specific transcriptome alteration should be directly addressed by single‐cell RNA‐seq analyses in the future.

In summary, the current study provided the first transcriptomic landscape of gene expression in 10 tissues of normal mice with or without metformin treatment. These results revealed that metformin deeply impacted gene expression profile to exert both beneficial and deleterious effects in various healthy tissues, which helps a lot to evaluate the potential impact of metformin on their functions. Our findings revealed that long‐term treatment of metformin will cause hypertension under normal condition. Clearly, cautions should be taken when metformin is potentially prescribed to healthy individual for the purposes of reducing bodyweight control, anti‐ageing and prolonging lifespan.

## CONFLICT OF INTEREST

The authors declare that they have no conflicts of interest.

## AUTHOR CONTRIBUTION

**Yuhong Meng:** Investigation (lead); Validation (equal); Writing‐original draft (supporting). **Rui Xiang:** Investigation (equal); Validation (equal). **Han Yan:** Investigation (equal); Validation (equal). **Yiran Zhou:** Formal analysis (supporting); Validation (equal). **Yuntao Hu:** Investigation (supporting). **Jichun Yang:** Conceptualization (lead); Supervision (lead); Writing‐review & editing (equal). **Yuan Zhou:** Formal analysis (lead); Supervision (supporting); Writing‐original draft (lead). **Qinghua Cui:** Conceptualization (lead); Supervision (lead); Writing‐review & editing (lead).

## ETHICAL APPROVAL

All animal experimental protocols complied with the Animal Management Rules of the Ministry of Health of the People's Republic of China and the Guide for the Care and Use of the Laboratory Animals of Peking University.

## Supporting information

Supplementary Material

## Data Availability

The transcriptome data of this study have been deposited in the GEO database (https://www.ncbi.nlm.nih.gov/geo/) under ID code GSE90755. The raw reads have been also deposited in the GSA database (http://bigd.big.ac.cn/gsa/) under ID code CRA000359.

## References

[1] Pernicova I , Korbonits M . Metformin–mode of action and clinical implications for diabetes and cancer. Nat Rev Endocrinol. 2014;10:143‐156. 24393785 10.1038/nrendo.2013.256

[2] Miller RA , Chu Q , Xie J , Foretz M , Viollet B , Birnbaum MJ . Biguanides suppress hepatic glucagon signalling by decreasing production of cyclic AMP. Nature. 2013;494:256‐260. 23292513 10.1038/nature11808 PMC3573218

[3] Johanns M , Lai YC , Hsu MF , et al. AMPK antagonizes hepatic glucagon‐stimulated cyclic AMP signalling via phosphorylation‐induced activation of cyclic nucleotide phosphodiesterase 4B. Nat Commun. 2016;7:10856. 26952277 10.1038/ncomms10856 PMC4786776

[4] Hunter RW , Hughey CC , Lantier L , et al. Metformin reduces liver glucose production by inhibition of fructose‐1‐6‐bisphosphatase. Nat Med. 2018;24:1395‐1406. 30150719 10.1038/s41591-018-0159-7 PMC6207338

[5] Madiraju AK , Qiu Y , Perry RJ , et al. Metformin inhibits gluconeogenesis via a redox‐dependent mechanism in vivo. Nat Med. 2018;24:1384‐1394. 30038219 10.1038/s41591-018-0125-4 PMC6129196

[6] Apaijai N , Pintana H , Chattipakorn SC , Chattipakorn N . Cardioprotective effects of metformin and vildagliptin in adult rats with insulin resistance induced by a high‐fat diet. Endocrinology. 2012;153:3878‐3885. 22621958 10.1210/en.2012-1262

[7] Holman RR , Paul SK , Bethel MA , Matthews DR , Neil HA . 10‐year follow‐up of intensive glucose control in type 2 diabetes. N Engl J Med. 2008;359:1577‐1589. 18784090 10.1056/NEJMoa0806470

[8] Petrie JR , Chaturvedi N , Ford I , et al. Cardiovascular and metabolic effects of metformin in patients with type 1 diabetes (REMOVAL): a double‐blind, randomised, placebo‐controlled trial. Lancet Diabetes Endocrinol. 2017;5:597‐609. 28615149 10.1016/S2213-8587(17)30194-8 PMC5641446

[9] Higurashi T , Hosono K , Takahashi H , et al. Metformin for chemoprevention of metachronous colorectal adenoma or polyps in post‐polypectomy patients without diabetes: a multicentre double‐blind, placebo‐controlled, randomised phase 3 trial. Lancet Oncol. 2016;17:475‐483. 26947328 10.1016/S1470-2045(15)00565-3

[10] Yousef M , Tsiani E . Metformin in lung cancer: review of in vitro and in vivo animal studies. Cancers (Basel). 2017;9:pii: E45. 10.3390/cancers9050045 PMC5447955 28481268

[11] Gong J , Robbins LA , Lugea A , Waldron RT , Jeon CY , Pandol SJ . Diabetes, pancreatic cancer, and metformin therapy. Front Physiol. 2014;5:426. 25426078 10.3389/fphys.2014.00426 PMC4224068

[12] Sonnenblick A , Agbor‐Tarh D , Bradbury I , et al. Impact of diabetes, insulin, and metformin use on the outcome of patients with human epidermal growth factor receptor 2‐positive primary breast cancer: analysis from the ALTTO phase III randomized trial. J Clin Oncol. 2017;35:1421‐1429. 28375706 10.1200/JCO.2016.69.7722 PMC5455460

[13] Bednarska S , Siejka A . The pathogenesis and treatment of polycystic ovary syndrome: What's new? Adv Clin Exp Med. 2017;26:359‐367. 28791858 10.17219/acem/59380

[14] Markowicz‐Piasecka M , Sikora J , Szydlowska A , Skupien A , Mikiciuk‐Olasik E , Huttunen KM . Metformin ‐ a future therapy for neurodegenerative diseases: theme: drug discovery, development and delivery in Alzheimer's disease guest editor: Davide Brambilla. Pharm Res. 2017;34:2614‐2627. 28589443 10.1007/s11095-017-2199-y PMC5736777

[15] Rangarajan S , Bone NB , Zmijewska AA , et al. Metformin reverses established lung fibrosis in a bleomycin model. Nat Med. 2018;24:1121‐1131. 29967351 10.1038/s41591-018-0087-6 PMC6081262

[16] Negrotto L , Farez MF , Correale J . Immunologic effects of metformin and pioglitazone treatment on metabolic syndrome and multiple sclerosis. JAMA Neurol. 2016;73:520‐528. 26953870 10.1001/jamaneurol.2015.4807

[17] Gantois I , Khoutorsky A , Popic J , et al. Metformin ameliorates core deficits in a mouse model of fragile X syndrome. Nat Med. 2017;23:674‐677. 28504725 10.1038/nm.4335

[18] Liu X , Chhipa RR , Pooya S , et al. Discrete mechanisms of mTOR and cell cycle regulation by AMPK agonists independent of AMPK. Proc Natl Acad Sci U S A. 2014;111:E435‐E444. 24474794 10.1073/pnas.1311121111 PMC3910576

[19] Cameron AR , Morrison VL , Levin D , et al. Anti‐inflammatory effects of metformin irrespective of diabetes status. Circ Res. 2016;119:652‐665. 27418629 10.1161/CIRCRESAHA.116.308445 PMC4990459

[20] Kulkarni AS , Brutsaert EF , Anghel V , et al. Metformin regulates metabolic and nonmetabolic pathways in skeletal muscle and subcutaneous adipose tissues of older adults. Aging Cell. 2018;17(2):e12723. 10.1111/acel.12723 PMC5847877 29383869

[21] Martin‐Montalvo A , Mercken EM , Mitchell SJ , et al. Metformin improves healthspan and lifespan in mice. Nat Commun. 2013;4:2192. 23900241 10.1038/ncomms3192 PMC3736576

[22] Castillo‐Quan JI , Blackwell TK . Metformin: restraining nucleocytoplasmic shuttling to fight cancer and aging. Cell. 2016;167:1670‐1671. 27984715 10.1016/j.cell.2016.11.058

[23] Barzilai N , Crandall JP , Kritchevsky SB , Espeland MA . Metformin as a tool to target aging. Cell Metab. 2016;23:1060‐1065. 27304507 10.1016/j.cmet.2016.05.011 PMC5943638

[24] Jensen JB , Sundelin EI , Jakobsen S , et al. [11C]‐labeled metformin distribution in the liver and small intestine using dynamic positron emission tomography in mice demonstrates tissue‐specific transporter dependency. Diabetes. 2016;65:1724‐1730. 26993065 10.2337/db16-0032

[25] Tartarin P , Moison D , Guibert E , et al. Metformin exposure affects human and mouse fetal testicular cells. Hum Reprod. 2012;27:3304‐3314. 22811314 10.1093/humrep/des264

[26] Hanem LGE , Stridsklev S , Juliusson PB , et al. Metformin use in PCOS pregnancies increases the risk of offspring overweight at 4 years of age: follow‐up of two RCTs. J Clin Endocrinol Metab. 2018;103:1612‐1621. 29490031 10.1210/jc.2017-02419

[27] Liang J , Mills GB . AMPK: a contextual oncogene or tumor suppressor? Cancer Res. 2013;73:2929‐2935. 23644529 10.1158/0008-5472.CAN-12-3876 PMC3725287

[28] DeCensi A , Puntoni M , Gandini S , et al. Differential effects of metformin on breast cancer proliferation according to markers of insulin resistance and tumor subtype in a randomized presurgical trial. Breast Cancer Res Treat. 2014;148:81‐90. 25253174 10.1007/s10549-014-3141-1 PMC4196136

[29] Jeon SM , Chandel NS , Hay N . AMPK regulates NADPH homeostasis to promote tumour cell survival during energy stress. Nature. 2012;485:661‐665. 22660331 10.1038/nature11066 PMC3607316

[30] Forslund K , Hildebrand F , Nielsen T , et al. Disentangling type 2 diabetes and metformin treatment signatures in the human gut microbiota. Nature. 2015;528:262‐266. 26633628 10.1038/nature15766 PMC4681099

[31] Cani PD , Van Hul M , Lefort C , Depommier C , Rastelli M , Everard A . Microbial regulation of organismal energy homeostasis. Nature Metabolism. 2019;1:34‐46. 10.1038/s42255-018-0017-4 32694818

[32] Sun L , Xie C , Wang G , et al. Gut microbiota and intestinal FXR mediate the clinical benefits of metformin. Nat Med. 2018;24:1919‐1929. 30397356 10.1038/s41591-018-0222-4 PMC6479226

[33] Ma W , Chen J , Meng Y , Yang J , Cui Q , Zhou Y . Metformin alters gut microbiota of healthy mice: implication for its potential role in gut microbiota homeostasis. Front Microbiol. 2018;9:1336. 29988362 10.3389/fmicb.2018.01336 PMC6023991

[34] Mullard A . Anti‐ageing pipeline starts to mature. Nat Rev Drug Discov. 2018;17:609‐612. 30072728 10.1038/nrd.2018.134

[35] Diabetes Prevention Program Research Group . Long‐term effects of lifestyle intervention or metformin on diabetes development and microvascular complications over 15‐year follow‐up: the Diabetes Prevention Program Outcomes Study. Lancet Diabetes Endocrinol. 2015;3:866‐875. 26377054 10.1016/S2213-8587(15)00291-0 PMC4623946

[36] Fahy GM , Brooke RT , Watson JP , et al. Reversal of epigenetic aging and immunosenescent trends in humans. Aging Cell. 2019;18(6):e13028. 31496122 10.1111/acel.13028 PMC6826138

[37] Konopka AR , Laurin JL , Schoenberg HM , et al. Metformin inhibits mitochondrial adaptations to aerobic exercise training in older adults. Aging Cell. 2019;18:e12880. 30548390 10.1111/acel.12880 PMC6351883

[38] Rattan R , Giri S , Hartmann LC , Shridhar V . Metformin attenuates ovarian cancer cell growth in an AMP‐kinase dispensable manner. J Cell Mol Med. 2011;15:166‐178. 19874425 10.1111/j.1582-4934.2009.00954.x PMC3822503

[39] Zhai L , Gu J , Yang D , Wang W , Ye S . Metformin ameliorates podocyte damage by restoring renal tissue podocalyxin expression in type 2 diabetic rats. J Diabetes Res. 2015;2015:231825. 26075281 10.1155/2015/231825 PMC4444588

[40] Hashimoto Y , Tanaka M , Okada H , et al. Postprandial hyperglycemia was ameliorated by taking metformin 30 min before a meal than taking metformin with a meal; a randomized, open‐label, crossover pilot study. Endocrine. 2016;52:271‐276. 26518190 10.1007/s12020-015-0786-4

[41] Howell JJ , Hellberg K , Turner M , et al. Metformin inhibits hepatic mTORC1 signaling via dose‐dependent mechanisms involving AMPK and the TSC complex. Cell Metab. 2017;25:463‐471. 28089566 10.1016/j.cmet.2016.12.009 PMC5299044

[42] Donadon V , Balbi M , Mas MD , Casarin P , Zanette G . Metformin and reduced risk of hepatocellular carcinoma in diabetic patients with chronic liver disease. Liver Int. 2010;30:750‐758. 20331505 10.1111/j.1478-3231.2010.02223.x

[43] Li J , Hernanda PY , Bramer WM , Peppelenbosch MP , van Luijk J , Pan Q . Anti‐tumor effects of metformin in animal models of hepatocellular carcinoma: a systematic review and meta‐analysis. PLoS One. 2015;10:e0127967. 26030161 10.1371/journal.pone.0127967 PMC4451077

[44] Mazza A , Fruci B , Garinis GA , Giuliano S , Malaguarnera R , Belfiore A . The role of metformin in the management of NAFLD. Exp Diabetes Res. 2012;2012:716404. 22194737 10.1155/2012/716404 PMC3238361

[45] Li H , Chen X , Yu Y , et al. Metformin inhibits the growth of nasopharyngeal carcinoma cells and sensitizes the cells to radiation via inhibition of the DNA damage repair pathway. Oncol Rep. 2014;32:2596‐2604. 25333332 10.3892/or.2014.3485

[46] Ferreira C , Sousa M , Rabaca A , Oliveira PF , Alves MG , Sa R . Impact of metformin on male reproduction. Curr Pharm Des. 2015;21:3621‐3633. 26166607 10.2174/1381612821666150710150041

[47] Guo J , Zhou Y , Cheng Y , et al. Metformin‐induced changes of the coding transcriptome and non‐coding RNAs in the livers of non‐alcoholic fatty liver disease mice. Cell Physiol Biochem. 2018;45:1487‐1505. 29466788 10.1159/000487575

[48] Hsu SY , Liou JW , Cheng TL , et al. beta‐Naphthoflavone protects from peritonitis by reducing TNF‐alpha‐induced endothelial cell activation. Pharmacol Res. 2015;102:192‐199. 26453957 10.1016/j.phrs.2015.10.001

[49] Woo SL , Xu H , Li H , et al. Metformin ameliorates hepatic steatosis and inflammation without altering adipose phenotype in diet‐induced obesity. PLoS One. 2014;9:e91111. 24638078 10.1371/journal.pone.0091111 PMC3956460

[50] Berstein LM . Metformin in obesity, cancer and aging: addressing controversies. Aging (Albany NY). 2012;4:320‐329. 22589237 10.18632/aging.100455 PMC3384433

[51] Smith Jr DL , Elam Jr CF , Mattison JA , et al. Metformin supplementation and life span in Fischer‐344 rats. J Gerontol A Biol Sci Med Sci. 2010;65:468‐474. 20304770 10.1093/gerona/glq033 PMC2854888

[52] Lamanna C , Monami M , Marchionni N , Mannucci E . Effect of metformin on cardiovascular events and mortality: a meta‐analysis of randomized clinical trials. Diabetes Obes Metab. 2011;13:221‐228. 21205121 10.1111/j.1463-1326.2010.01349.x

[53] Pedram A , Razandi M , Lubahn D , Liu J , Vannan M , Levin ER . Estrogen inhibits cardiac hypertrophy: role of estrogen receptor‐beta to inhibit calcineurin. Endocrinology. 2008;149:3361‐3369. 18372323 10.1210/en.2008-0133 PMC2453079

[54] Ryu JY , Kim HU , Lee SY . Deep learning improves prediction of drug‐drug and drug‐food interactions. Proc Natl Acad Sci USA. 2018;115:E4304‐E4311. 29666228 10.1073/pnas.1803294115 PMC5939113

[55] Wu RR , Zhang FY , Gao KM , et al. Metformin treatment of antipsychotic‐induced dyslipidemia: an analysis of two randomized, placebo‐controlled trials. Mol Psychiatry. 2016;21:1537‐1544. 26809842 10.1038/mp.2015.221 PMC5078852

[56] Henderson DC , Daley TB , Kunkel L , Rodrigues‐Scott M , Koul P , Hayden D . Clozapine and hypertension: a chart review of 82 patients. J Clin Psychiatry. 2004;65:686‐689. 15163256 10.4088/jcp.v65n0514

[57] Fang J , Yang J , Wu X , et al. Metformin alleviates human cellular aging by upregulating the endoplasmic reticulum glutathione peroxidase 7. Aging Cell. 2018;17:e12765. 29659168 10.1111/acel.12765 PMC6052468

[58] Dehghan E , Zhang Y , Saremi B , et al. Hydralazine induces stress resistance and extends C. elegans lifespan by activating the NRF2/SKN‐1 signalling pathway. Nat Commun. 2017;8:2223. 29263362 10.1038/s41467-017-02394-3 PMC5738364

[59] Gu J , Yin ZF , Zhang JF , Wang CQ . Association between long‐term prescription of metformin and the progression of heart failure with preserved ejection fraction in patients with type 2 diabetes mellitus and hypertension. Int J Cardiol. 2020;306:140‐145. 31711850 10.1016/j.ijcard.2019.11.087

[60] Derosa G , Fogari E , Cicero AF , et al. Blood pressure control and inflammatory markers in type 2 diabetic patients treated with pioglitazone or rosiglitazone and metformin. Hypertens Res. 2007;30:387‐394. 17587750 10.1291/hypres.30.387

[61] He H , Zhao Z , Chen J , et al. Metformin‐based treatment for obesity‐related hypertension: a randomized, double‐blind, placebo‐controlled trial. J Hypertens. 2012;30:1430‐1439. 22525206 10.1097/HJH.0b013e328353e249

[62] Thomopoulos C , Katsimagklis G , Makris T . Metformin and blood pressure lowering: a questioned association. J Hypertens. 2017;35:27‐28. 27902624 10.1097/HJH.0000000000001146

[63] Xu X , Li J , Zou J , et al. Association of germline variants in natural killer cells with tumor immune microenvironment subtypes, tumor‐infiltrating lymphocytes, immunotherapy response, clinical outcomes, and cancer risk. JAMA Netw Open. 2019;2:e199292.31483464

[64] Aran D , Hu Z , Butte AJ . xCell: digitally portraying the tissue cellular heterogeneity landscape. Genome Biol. 2017;18:220. 29141660 10.1186/s13059-017-1349-1 PMC5688663

