# Supplementary material for: Transcriptomic landscape profiling of metformin‐treated healthy mice: Implication for potential hypertension risk when prophylactically used
Source: J Cell Mol Med. 2020 Jun 11;24(14):8138–50. doi: 10.1111/jcmm.15472 (PMC7348147; doi:10.1111/jcmm.15472)
Supplement: Supplementary file 1 — Supplementary Material [file JCMM-24-8138-s001.docx]

**Supporting Information**

**Transcriptomic Landscape Profiling of Metformin-Treated Healthy Mice: Implication for Potential Hypertension Risk** **When Prophylactically Used**

Yuhong Meng^1,#^, Rui Xiang^1,#^, Han Yan^1,#^, Yiran Zhou^1,#^, Yuntao Hu^1^, Jichun Yang^1,*^, Yuan Zhou^1,*^, Qinghua Cui^1,*^

**Content**

1. Supplementary Text

1.1. Functional and disease associations of shared differentially expressed genes (DEGs) and module-exclusive DEGs

1.2. Potential protective and deleterious effects of metformin on healthy kidney, brown adipose, brain, and stomach

1.3. Potential protective and deleterious effects of metformin on healthy skeletal muscle and aorta

1.4. Potential protective and deleterious effects of metformin on healthy eye and testis

2. Supplementary Figures

3. Supplementary Tables

**Appendix S1**

**1.1. Functional and disease associations of shared differentially expressed genes (DEGs) and module-exclusive DEGs**

To be more precise, we further analyzed the disease gene sets and enriched pathways by using the DAVID functional enrichment analysis tool (https://david.ncifcrf.gov/). As intuitively expected, disease genes of type 2 diabetes are overrepresented, with DEGs spanning across nearly all of the tissues (Fig. 2D). Multiple related pathways are also enriched, including but not limited to chemical carcinogenesis, cytochrome P450 drug metabolism, linoleic acid metabolism, and PPAR signaling pathways. Likewise, the genes associated with chronic kidney failure and PPAR signaling pathway are also enriched in the shared DEGs, indicating the wide interplay between PPAR pathway and metformin. The disease genes of hypercholesterolemia and hyperlipidemia are at the top of the disease gene set list and are largely up-regulated by metformin in both brown adipose and liver, which are in line with the important roles of metformin in regulating lipid homeostasis. Besides, though not strongly linked to a specific disease gene set, the metabolic pathway about carbohydrate digestion and absorption, and that about starch and sucrose metabolism are also overrepresented. One interesting disease gene set is related to precocious puberty, which is also connected with steroid hormone biosynthesis and de-regulated in multiple tissues including testis. This observation again emphasizes the importance of assessing the metformin’s impact on sexual development. Another noticeable association is that with myocardial infarction disease genes. These genes are down-regulated in both aorta and heart. Whether this association implies the cardiovascular beneficial roles of metformin, or deleterious roles instead, will be assessed by physiology experiments presented in the last section.

We further clustered the tissues based on their correlation of DEGs (Fig. 2D). The aorta, brain and brown adipose, in which genes are largely up-regulated by metformin treatment, are grouped as one module (Mod_up). Similarly, the tissues where genes are largely down-regulated, including eye, liver and skeletal muscle, are grouped as another module (Mod_down). Finally, the rest tissues (stomach, heart, kidney, and testis) showing the DEG pattern in-between form a loosely connected module (Mod_inbetween). The Mod_up are enriched for the disease genes of multiple auto-immune diseases including type 1 diabetes, celiac disease, systematic lupus erythematosus, rheumatoid arthritis, and autoimmune hepatitis. These disease genes are also shared by the enriched pathways like allograft rejection pathway, human T-lymphotropic virus 1 (HTLV-I) infection pathway, and the pathways associated with graft-versus-host disease or autoimmune thyroid disease (Figure S2A). This result suggests potential wide immunoregulatory role of metformin. Some of these disease and pathway gene sets can also be found in the top list for Mod_down. For example, the disease genes of type 1 diabetes are enriched in exclusive DEGs of Mod_down, which is also related to the enriched pathways underlying graft-versus-host disease or autoimmune thyroid disease (Figure S2B). Nevertheless, given the overall negative correlation of DEGs between Mod_up and Mod_down, it is possible that the direction of influence of metformin on immunity is tissue-dependent. Besides, disease genes of viral hepatitis are also overrepresented, in line with the hepatoprotective role of metformin. Another noticeable term is myasthenia gravis which can lead to muscle weakness. The risk of metformin to induce weakness muscle should be assessed for long-term use on non-diabetic individuals. Finally, the exclusive DEGs from the loosely connected modules Mod_inbetween have also shown some particular disease gene associations (Figure S2C). Notably, the coronary heart disease and hypertension are presented in the top list of the enriched disease gene sets. Whether extended period of metformin treatment would induce cardiovascular abnormalities will be experimentally assessed in the last section of Results in the main text. We also tested the overrepresented transcription factors behind the DEGs from each module by using ChIP-Atlas platform. Th top 15 overrepresented transcription factors for Mod_up, Mod_down and Mod_inbetween are shown in Supplementary Figure S3A-C, respectively. Notably, some transcription factors are shared between modules, indicating common transcriptional regulation mechanisms underlying metformin’s beneficial and deleterious effects. Some of these transcription factors are well-studied in the regulation axis of metformin. For example, SMAD3 is known to be involved in the mechanisms of metformin action against obesity [1], liver fibrosis [2] and melanoma [3]. On the other hand, the relationship between metformin and other transcription factors like CHD8 and BRD2 have not been elucidated, and would be experimentally investigated to find novel mechanisms and target pathways of metformin.

**1.2. Potential protective and deleterious effects of metformin on** **healthy kidney, brown adipose, brain, and stomach**

In kidney, multiple beneficial roles of metformin are supported by the wide negative correlations between the metformin signature and disease model signatures (Figure S3A) including the VhL mutant model of renal clear cell adenocarcinoma (SCC = -0.137, FDR = 2.88E-66) and the D2.B6-Ins2Akita/MatbJ and OVE26 models of diabetic nephropathy (SCC = -0.168 and -0.138, FDR = 6.81E-96 and 1.19E-65). Interestingly, activation of PPAR pathway is implicated in ameliorating several kidney diseases [4] and the metformin signature positively correlates with the PPAR agonist treatment signature (SCC = 0.132, FDR = 8.84E-60), indicating metformin may raise protective action on kidney through the activation of PPAR pathway. One noticeable exception is that the metformin signature positively correlates with the diabetic nephropathy signatures in db/db mouse model of type 2 diabetes (Table 1; SCC = 0.115, FDR = 6.85E-41). Indeed, recent investigation on db/db mouse model has implied potential kidney risk of metformin treatment [5], recalling the concerns about the kidney safety of metformin [6].

According to the results of signature comparison analysis, the metformin signature in brown adipose, brain, and stomach are also prone to be beneficial. In brown adipose, metformin signature negatively correlates with signatures of both type 1 (NOD model) (SCC = -0.104, FDR = 1.02E-36) and type 2 (MKR model) (SCC = -0.076, FDR = 9.56E-19) diabetic disease models (Figure S3B). It also positively correlates with CL316243 treatment-induced white adipose browning signature (SCC = 0.141, FDR = 7.05E-65) and short-term calorie restriction signature (SCC = 0.112, FDR = 3.39E-44). It also shows significant similarities with narciclasine intervention against HFD-induced obesity (SCC = 0.114, FDR = 4.42E-40). These results are in line with the recent evidence in which the metformin exert beneficial role on lipid accumulation by promoting the brown adipose differentiation and related gene expression [7]. In brain (Figure S3C), metformin negatively correlates with several hazardous conditions including sleep deprivation (SCC = -0.148, FDR = 1.36E-77), Ercc1-/+ and the paraquat treatment induced Parkinson’s disease models (SCC = -0.160 and -0.143, FDR = 7.64E-84 and 3.39E-38), Shank2 e6-7 KO and BTBR models of autism spectrum disorder (SCC = -0.142 and -0.115, FDR = 2.67E-72 and 1.89E-28) and abuses of morphine, nicotine, heroin and cocaine (SCC = -0.093, -0.063, -0.061 and -0.060, FDR = 2.81E-24, 2.40E-10, 6.33E-10 and 1.66E-9). Indeed, recent study has suggested metformin intervention could partly reverse the mitochondrion abnormality in Parkinson disease model [8]. In stomach (Figure S3D), the metformin signature negatively correlates with high fat diet induced obesity (SCC = -0.082, FDR = 1.19E-22) and dexamethasone-induced gastroparesis (SCC = -0.078, FDR = 9.26E-22). On the other hand, however, it also correlates with disease model signatures of acetic acid treatment induced stomach ulcer (SCC = 0.168, FDR = 3.72E-103) and Wnt1/C2mE over expression model of gastric adenocarcinoma (SCC = 0.068, FDR = 3.61E-17). Indeed, metformin’s adverse side effect on stomach is commonly reported [9], while its efficacy on gastric adenocarcinoma in non-diabetic population is still unclear [10]. Thus, the potential deleterious effect of metformin on healthy stomach seems also noteworthy.

**1.3. Potential protective and deleterious effects of metformin on healthy skeletal muscle and aorta**

In skeletal muscle, the metformin signature turns out to be much more deleterious than background (Figure S4A). The metformin signature positively correlates with fasting signature (SCC = 0.131, FDR = 5.13E-60) and the potential anti-aging drug nicotinamide mononucleotide signature (SCC = 0.132, FDR = 1.85E-37), which recapitulates the previous observations of the anti-aging effect of metformin in muscle by mimicking caloric restriction [11]. On the other hand, however, metformin signature also correlates with multiple skeletal muscle atrophy or dystrophy models including but not limited to AR113Q-KRKR model of spinobulbar muscular atrophy (SCC = 0.186, FDR = 1.63E-105), tenotomy-induced muscle atrophy (SCC = 0.170, FDR = 1.74E-99), Lama2-/- model of congenital muscular dystrophy type 1A (SCC = 0.147, FDR = 1.77E-74) and hindlimb casting induced muscle atrophy (SCC = 0.129, FDR = 1.73E-56). Therefore, the impact of metformin on muscle functionality should be monitored when it is applied for long-term use on healthy population.

Besides, our comparative analysis in aorta (Figure S4B) indicates that the metformin signature also positively correlates with multiple deleterious signatures like high fat diet-induced atherosclerotic plaque (SCC = 0.281, FDR = 3.49E-275) and porcine pancreatic elastase (SCC = 0.180, FDR = 9.85E-115) or angiotensin II induced abdominal aortic aneurysm model (SCC = 0.099, FDR = 2.13E-35). Functional enrichment analysis of genes up-regulated by metformin in aorta suggests significant overrepresentation of genes involved in positive regulation of leukocyte cell-cell adhesion (FDR = 3.27E-9), regulation of mononuclear cell proliferation (FDR = 2.33E-7) and interferon-gamma production (FDR = 9.44E-5). It is well-documented that the diabetes negatively correlates with abdominal aortic aneurysm [12], and metformin may cancel the protective effect of diabetes through the deregulation of proteins responsible for vascular inflammation. Nevertheless, there are controversies about the associations between metformin treatment and abdominal aortic aneurysm [13], and the aorta transcriptome profiles exhibit high heterogenicity in our study (Figure S1). Thus, careful experimental validation is required to interpret these observations.

**1.4. Potential protective and deleterious effects of metformin on healthy eye and testis**

The results seem complicated in eye and testis. Metformin signature negatively correlates with db/db model of diabetic retinopathy (SCC = -0.108, FDR = 1.07E-45), E168D2 model of CRX-associated retinopathies (SCC = -0.102, FDR = 5.91E-26) and chir99021 induced retinal damage (SCC = -0.133, FDR = 4.94E-65) in eye (Figure S4C), suggesting potential beneficial roles. Whereas, it also negatively correlates with the signature of anti-inflammatory drugs dexamethasone (SCC = -0.280, FDR = 2.08E-294) but positively correlates with Rhod-/- (SCC = 0.094, FDR = 3.24E-32) and rd1 (SCC = 0.081, FDR = 2.81E-24) disease models of retinitis pigmentosa. Therefore, whether metformin would dysregulate eye inflammation responses requires further verification. Finally, in testis, the direction of correlation between metformin signature and male infertility signatures are disease model-dependent (Figure S4D). Nevertheless, as the application of metformin on young people is increasing, the impact of metformin on male fertility and sexual development should be carefully evaluated.

**References**

1. **Luo T, Nocon A, Fry J, Sherban A, Rui X, Jiang B, Xu XJ, Han J, Yan Y, Yang Q, Li Q, Zang M.** AMPK Activation by Metformin Suppresses Abnormal Extracellular Matrix Remodeling in Adipose Tissue and Ameliorates Insulin Resistance in Obesity. *Diabetes*. 2016; 65: 2295-310.

2. **Fan K, Wu K, Lin L, Ge P, Dai J, He X, Hu K, Zhang L.** Metformin mitigates carbon tetrachloride-induced TGF-beta1/Smad3 signaling and liver fibrosis in mice. *Biomed Pharmacother*. 2017; 90: 421-6.

3. **Li K, Zhang TT, Wang F, Cui B, Zhao CX, Yu JJ, Lv XX, Zhang XW, Yang ZN, Huang B, Li X, Hua F, Hu ZW.** Metformin suppresses melanoma progression by inhibiting KAT5-mediated SMAD3 acetylation, transcriptional activity and TRIB3 expression. *Oncogene*. 2018; 37: 2967-81.

4. **Ruan X, Zheng F, Guan Y.** PPARs and the kidney in metabolic syndrome. *Am J Physiol Renal Physiol*. 2008; 294: F1032-47.

5. **Zheng S, Liu J, Han Q, Huang S, Su W, Fu J, Jia X, Du S, Zhou Y, Zhang X, Guan Y.** Metformin induces renal medullary interstitial cell apoptosis in type 2 diabetic mice. *J Diabetes*. 2014; 6: 132-46.

6. **Lazarus B, Wu A, Shin JI, Sang Y, Alexander GC, Secora A, Inker LA, Coresh J, Chang AR, Grams ME.** Association of Metformin Use With Risk of Lactic Acidosis Across the Range of Kidney Function: A Community-Based Cohort Study. *JAMA Intern Med*. 2018; 178: 903-10.

7. **Kim EK, Lee SH, Lee SY, Kim JK, Jhun JY, Na HS, Kim SY, Choi JY, Yang CW, Park SH, Cho ML.** Metformin ameliorates experimental-obesity-associated autoimmune arthritis by inducing FGF21 expression and brown adipocyte differentiation. *Exp Mol Med*. 2018; 50: e432.

8. **Fitzgerald JC, Zimprich A, Carvajal Berrio DA, Schindler KM, Maurer B, Schulte C, Bus C, Hauser AK, Kubler M, Lewin R, Bobbili DR, Schwarz LM, Vartholomaiou E, Brockmann K, Wust R, Madlung J, Nordheim A, Riess O, Martins LM, Glaab E, May P, Schenke-Layland K, Picard D, Sharma M, Gasser T, Kruger R.** Metformin reverses TRAP1 mutation-associated alterations in mitochondrial function in Parkinson's disease. *Brain*. 2017; 140: 2444-59.

9. **Law V, Knox C, Djoumbou Y, Jewison T, Guo AC, Liu Y, Maciejewski A, Arndt D, Wilson M, Neveu V, Tang A, Gabriel G, Ly C, Adamjee S, Dame ZT, Han B, Zhou Y, Wishart DS.** DrugBank 4.0: shedding new light on drug metabolism. *Nucleic Acids Res*. 2014; 42: D1091-7.

10. **Sui X, Xu Y, Wang X, Han W, Pan H, Xiao M.** Metformin: A Novel but Controversial Drug in Cancer Prevention and Treatment. *Mol Pharm*. 2015; 12: 3783-91.

11. **Martin-Montalvo A, Mercken EM, Mitchell SJ, Palacios HH, Mote PL, Scheibye-Knudsen M, Gomes AP, Ward TM, Minor RK, Blouin MJ, Schwab M, Pollak M, Zhang Y, Yu Y, Becker KG, Bohr VA, Ingram DK, Sinclair DA, Wolf NS, Spindler SR, Bernier M, de Cabo R.** Metformin improves healthspan and lifespan in mice. *Nat Commun*. 2013; 4: 2192.

12. **De Rango P, Farchioni L, Fiorucci B, Lenti M.** Diabetes and abdominal aortic aneurysms. *Eur J Vasc Endovasc Surg*. 2014; 47: 243-61.

13. **Kristensen KL, Pottegard A, Hallas J, Rasmussen LM, Lindholt JS.** Metformin treatment does not affect the risk of ruptured abdominal aortic aneurysms. *J Vasc Surg*. 2017; 66: 768-74 e2.

**2. Supplementary Figures**

**
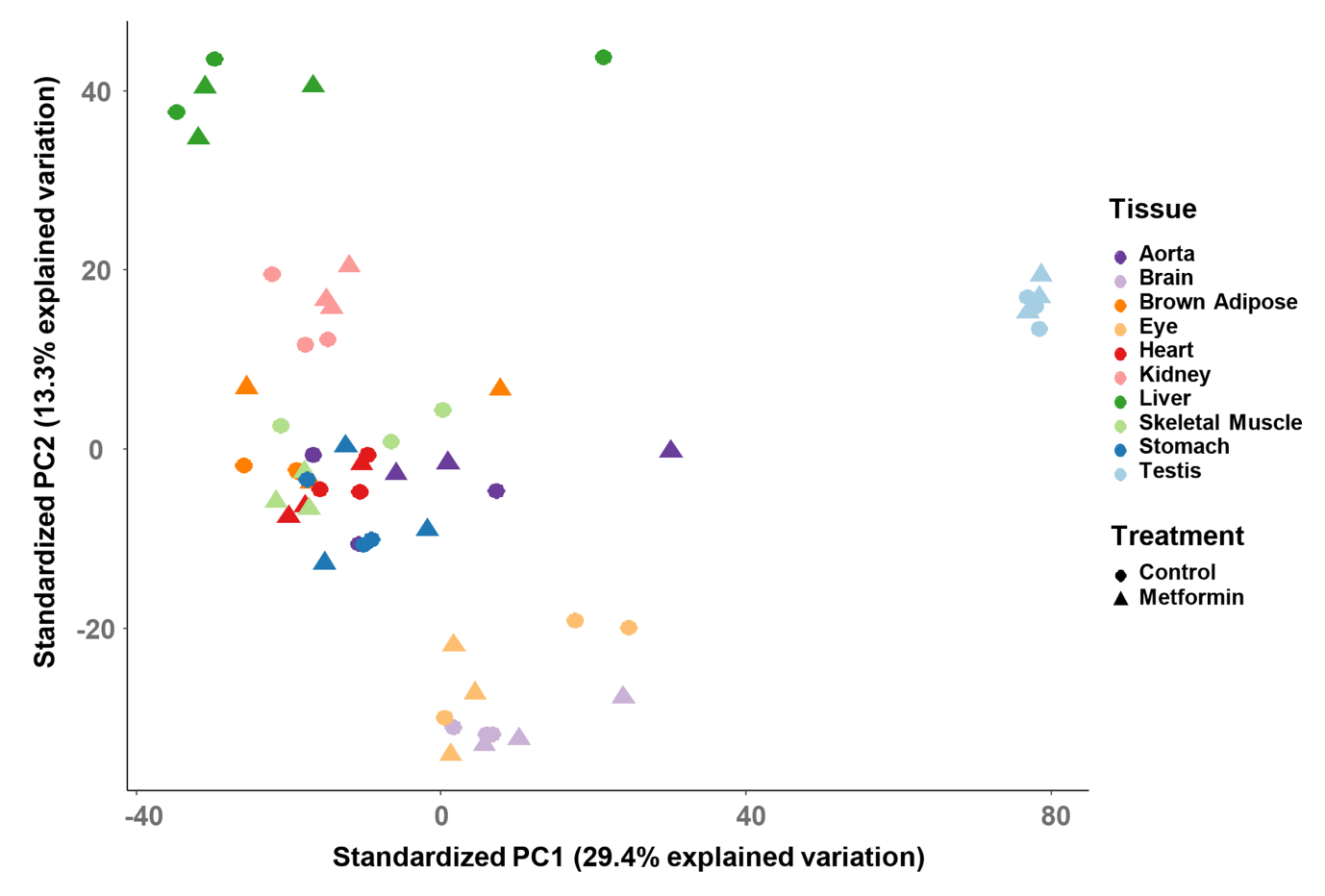
**

**Supplementary Figure S1. Principle component analysis (PCA) of the transcriptome profiles.**

The sample are plotted according to the first two principle components from the analysis of the transcriptome profiles of differentially expressed genes.


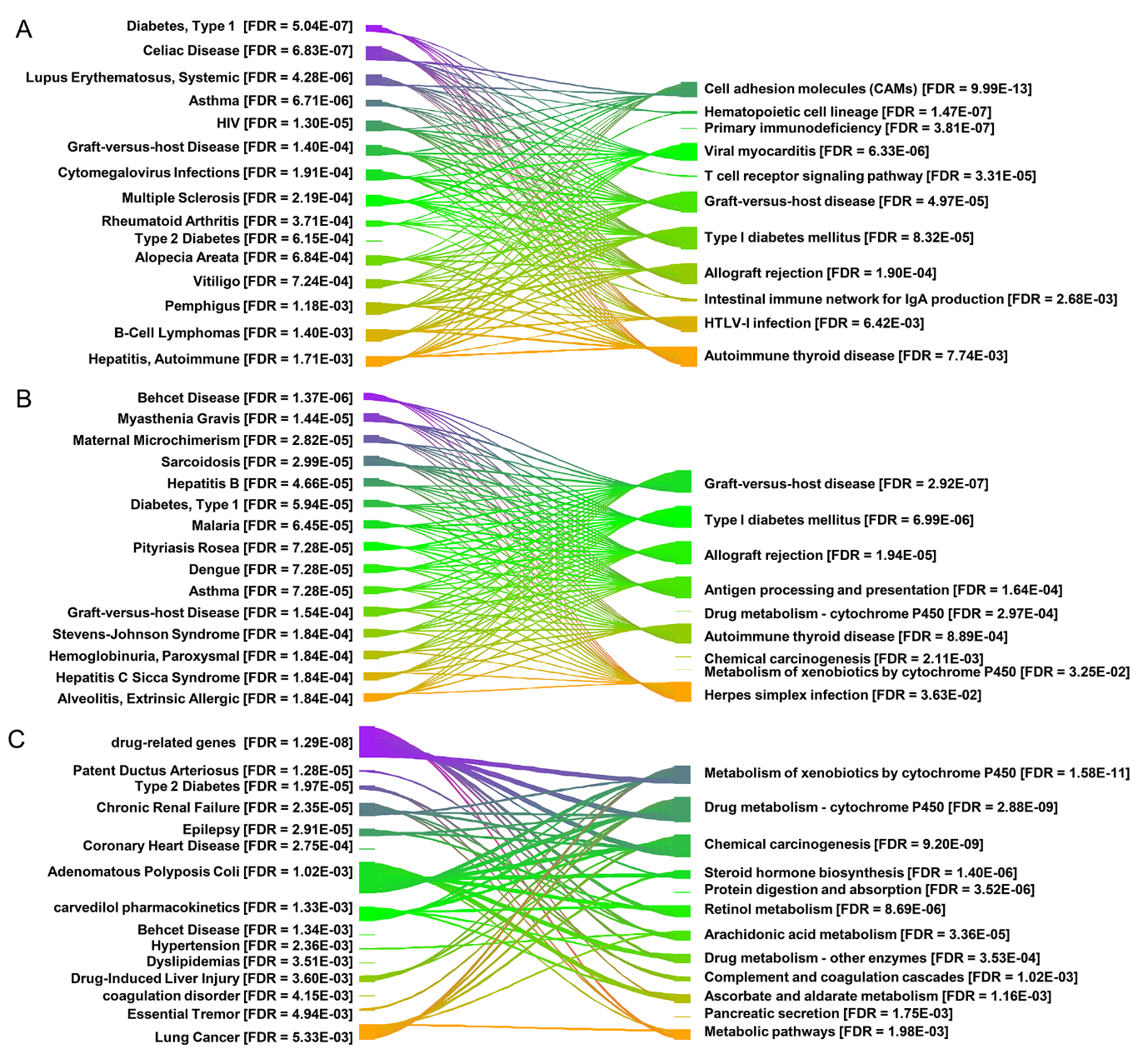


**Supplementary Figure S2. The GAD disease gene and KEGG pathway enrichment of the module-exclusive DEGs.**

The functional enrichment analysis results for DEGs exclusive among the (A) Mod_up, (B) Mod_down and (C) Mod_inbetween are shown. The redundant GAD disease gene sets are not shown. The width of the strap between GAD and KEGG terms correlates with the number of shared genes between them. Only straps indicating significant overlaps (Fisher exact test, P < 0.05) are shown.

**
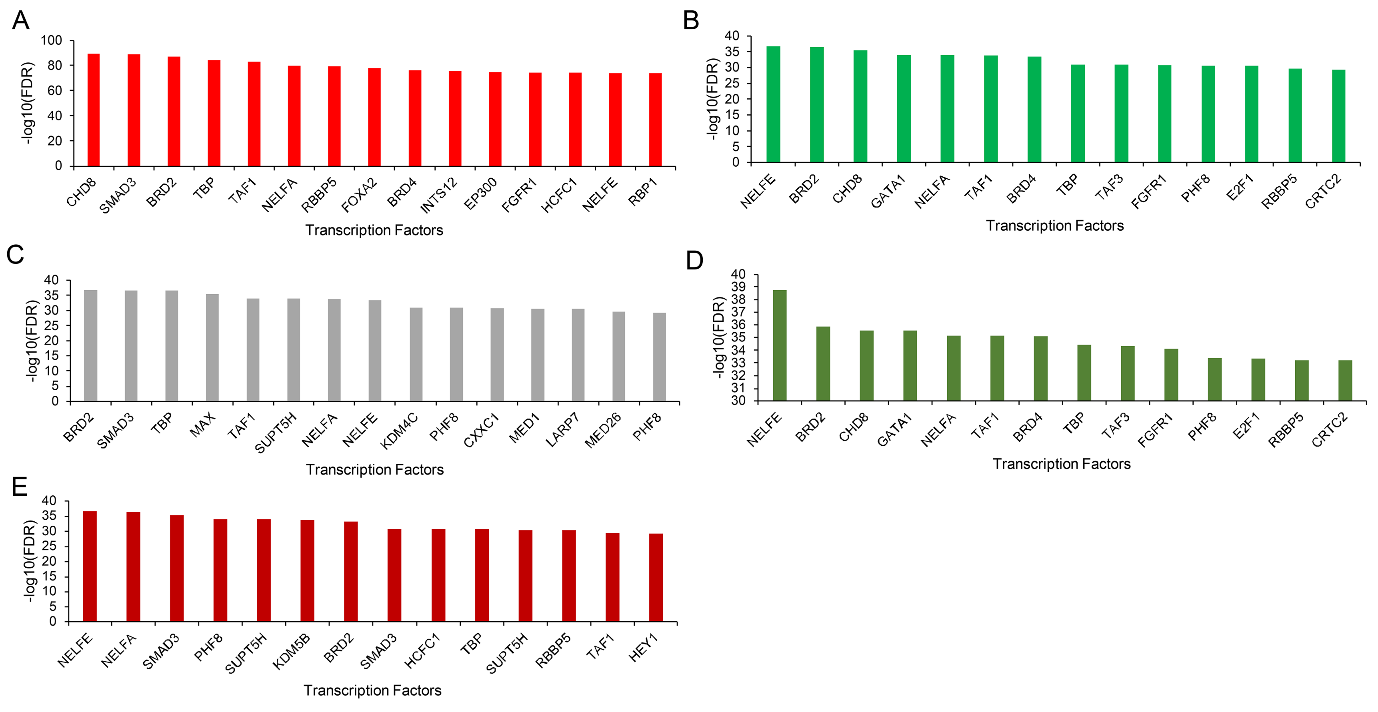
**

**Supplementary Figure S3. The top 15 overrepresented transcription factors behind the DEGs.** The top 15 overrepresented transcription factors behind the exclusive DEGs among the (A) Mod_up, (B) Mod_down and (C) Mod_inbetween, and among the DEGs in (D) liver and (E) heart are shown.

**
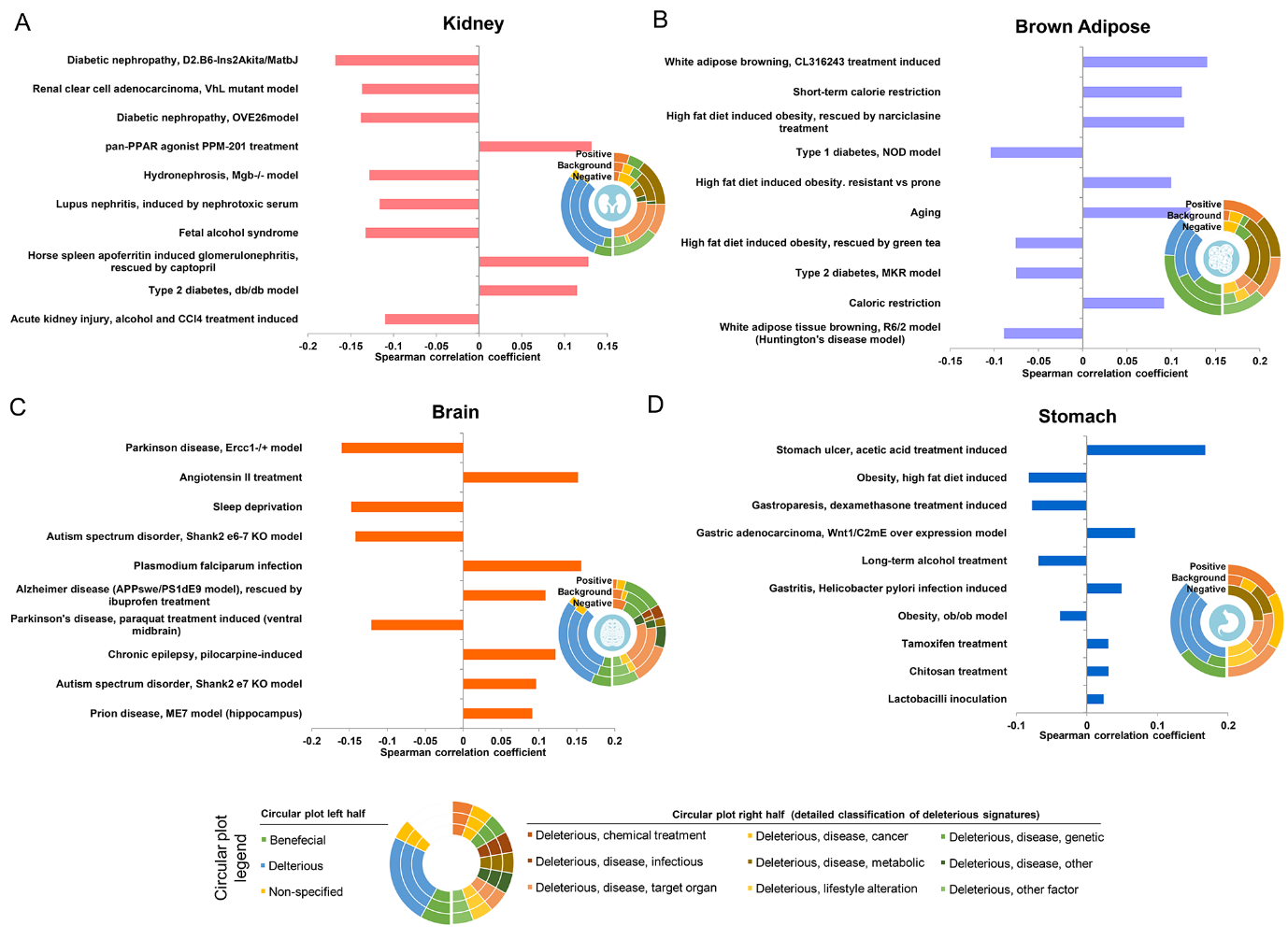
**

**Supplementary Figure S4. The significant correlations between metformin’s transcriptomic signature and curated reference transcriptomic signatures in kidney, brown adipose, brain and stomach**

The top 10 most significant correlation of transcriptomic signature (gene expression fold changes) between metformin-treated normal mice and other treatments or disease models are shown. (A) kidney, (B) brown adipose, (C) brain, (D) stomach.


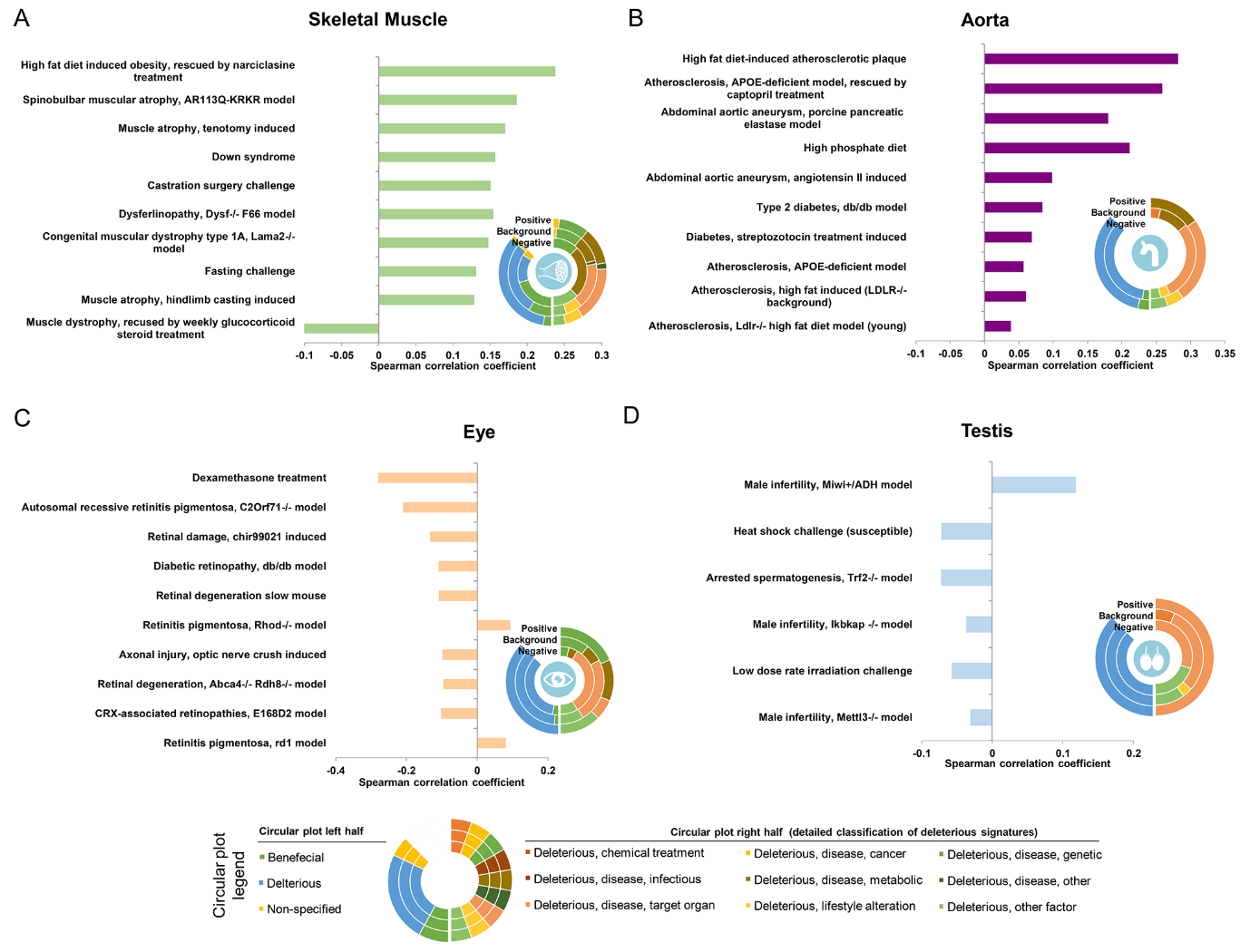


**Supplementary Figure S5. The significant correlations between metformin’s transcriptomic signature and curated reference transcriptomic signatures in skeletal muscle, aorta, eye and testis.**

The top 10 most significant correlation of transcriptomic signature (gene expression fold changes) between metformin-treated normal mice and other treatments or disease models are shown. (A) skeletal muscle, (B) aorta, (C) eye, (D) testis.

**
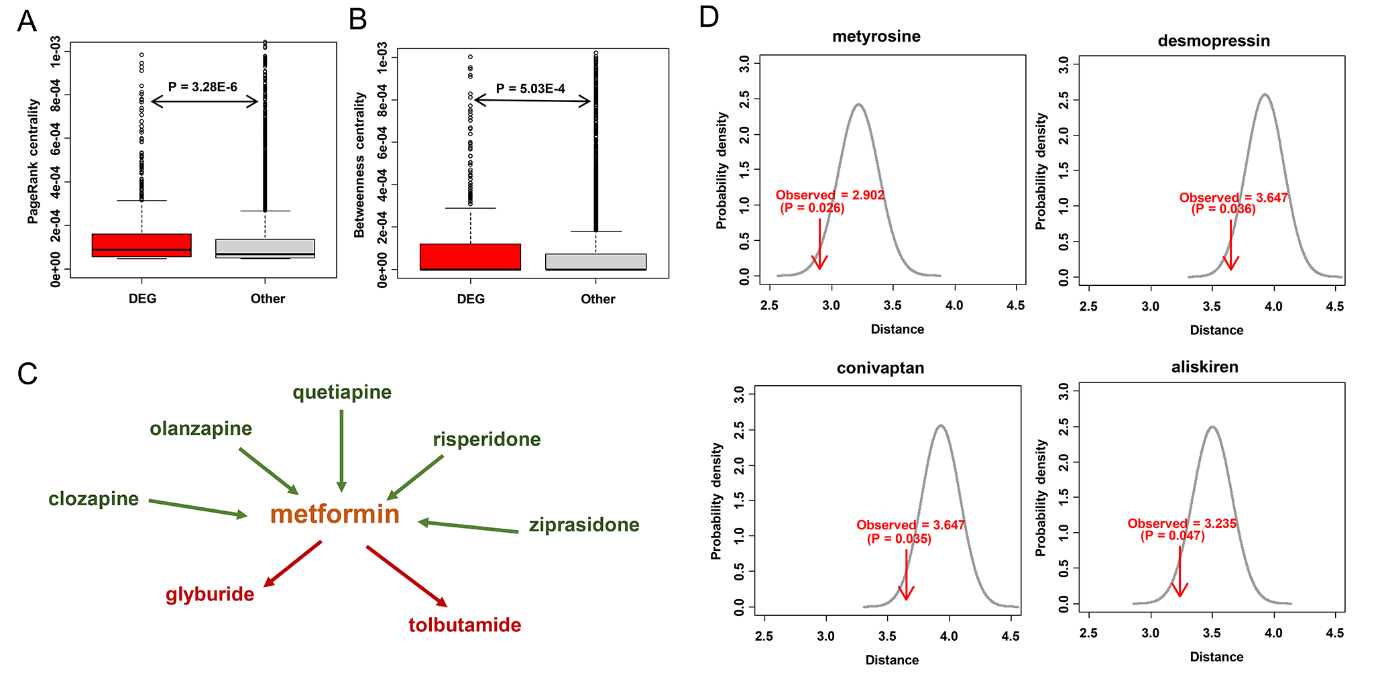
**

**Supplementary Figure S6. Topology analysis of DEGs and its relationship with other drugs and drug targets in human signaling network.**

(A) The comparison of PageRank centrality between DEGs and other genes in human signaling network. (B) The comparison of betweenness centrality between DEGs and other genes in human signaling network. (C) The DrugBank reported drug-drug interactions between metformin and the drugs that show potential functional links with metformin in the network analysis. Arrow from a green node indicates an interaction that suppresses the efficacy of drug shown at the target side of arrow, while arrow to a red node indicates an interaction that enhances the efficacy. (D) The sample comparisons between the observed distance between DEGs and the drug targets (red arrow), and those between DEGs and randomized targets (grey curve).

**
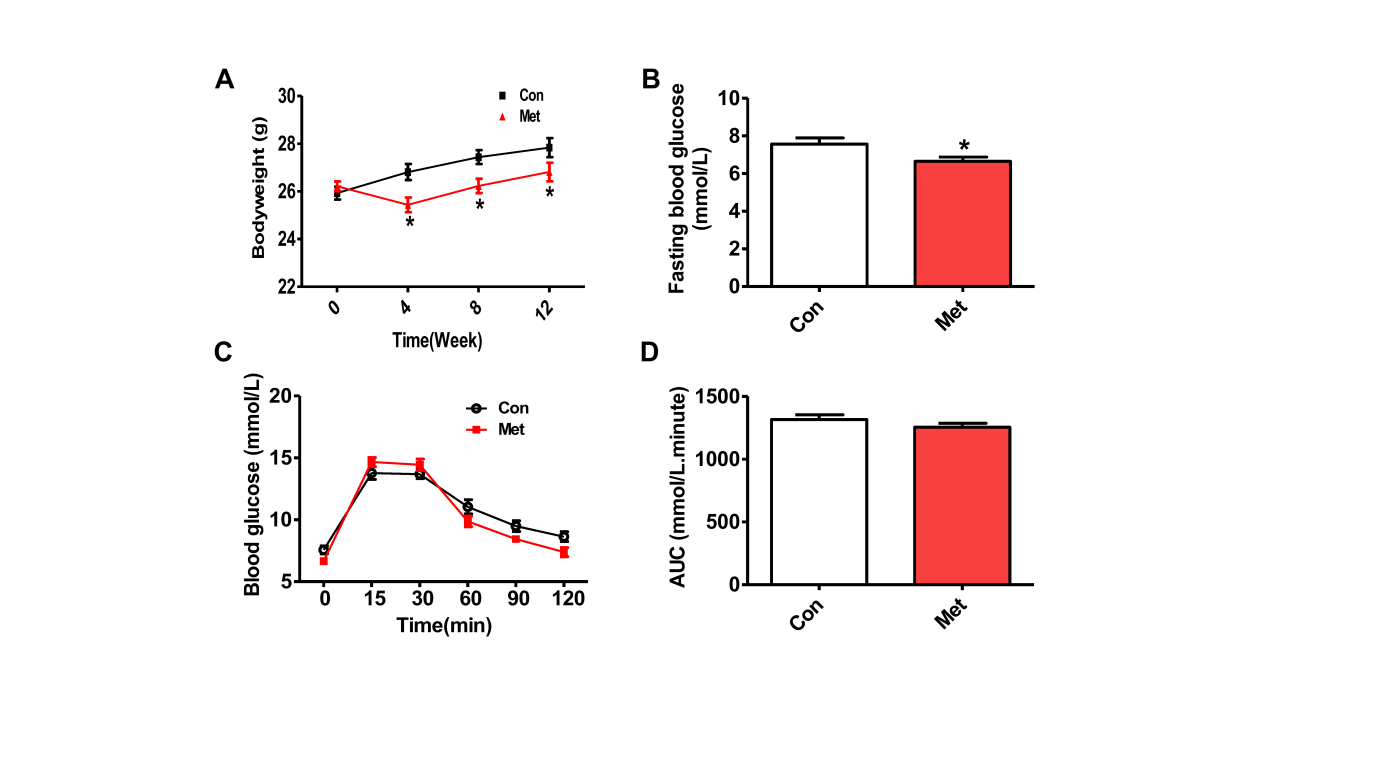
**

**Supplementary Figure S7. Long-term administration of metformin on blood glucose levels of young male C57BL/C mice**. 8-10 week old male normal C57BL/C mice were orally administrated with metformin (300 mg/kg body weight) for 3 months as described in experimental procedure. (A) The bodyweight of mice during treatment with metformin or water. (B) Fasting blood glucose levels of mice after treatment with metformin or water. (C) OGTT of mice after treatment with metformin or water. (D) AUC of OGTT data presented in panel C. N=10-12. The results are presented as the mean±SEM. Statistical significance of differences between groups was analyzed by t-test. *P<0.05 versus control group of mice.


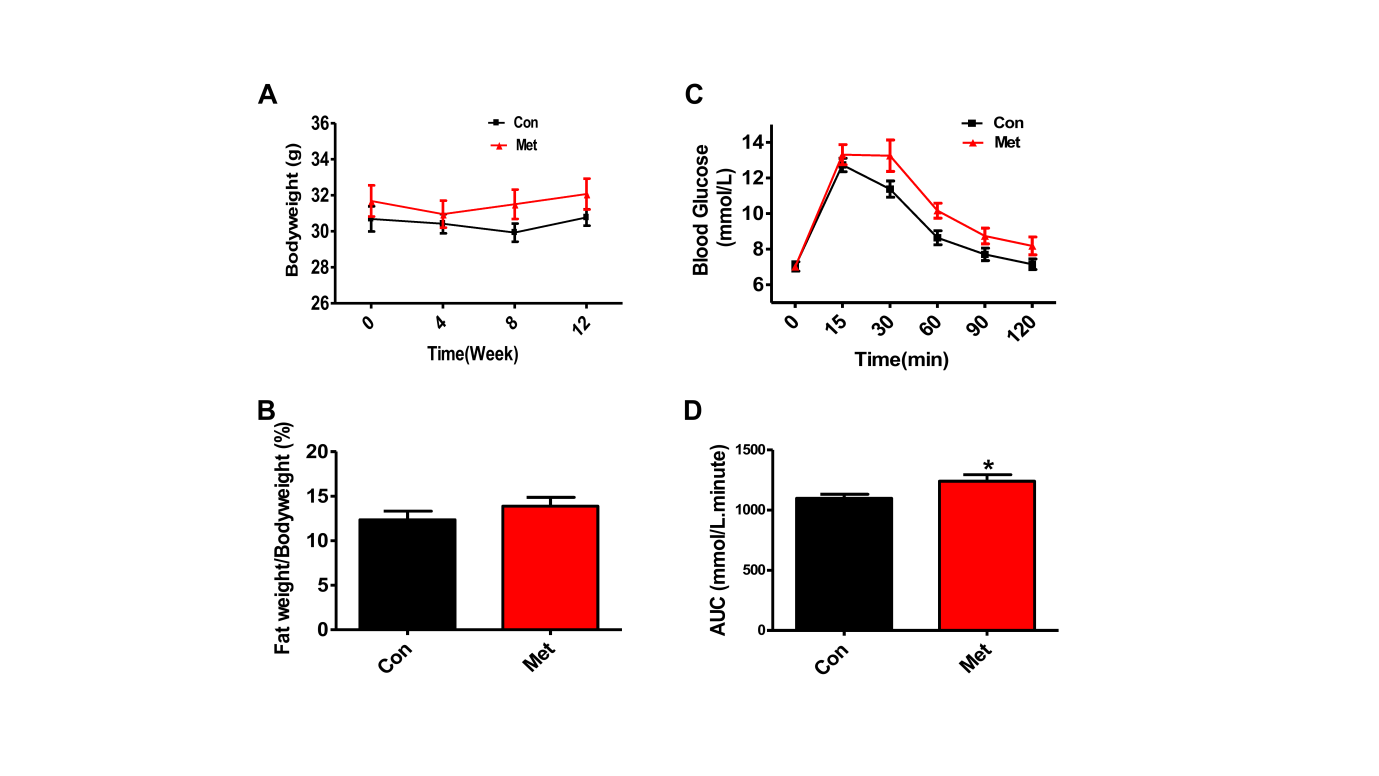


**Supplementary Figure S8. Long-term administration of metformin on blood glucose levels of old male C57BL/C mice**. 60-62 week old male normal C57BL/C mice were orally administrated with metformin (300 mg/kg body weight) for 3 months as described in experimental procedure. (A) The bodyweight of mice during the treatment with metformin or water. (B) The ration of white adipose weight to bodyweight after 3-month treatment. (C) OGTT of mice after 3-month treatment with metformin or water. (D) AUC of OGTT data presented in panel C. N=9, The results are presented as the mean±SEM. Statistical significance of differences between groups was analyzed by t-test. *P<0.05 versus control group of mice.


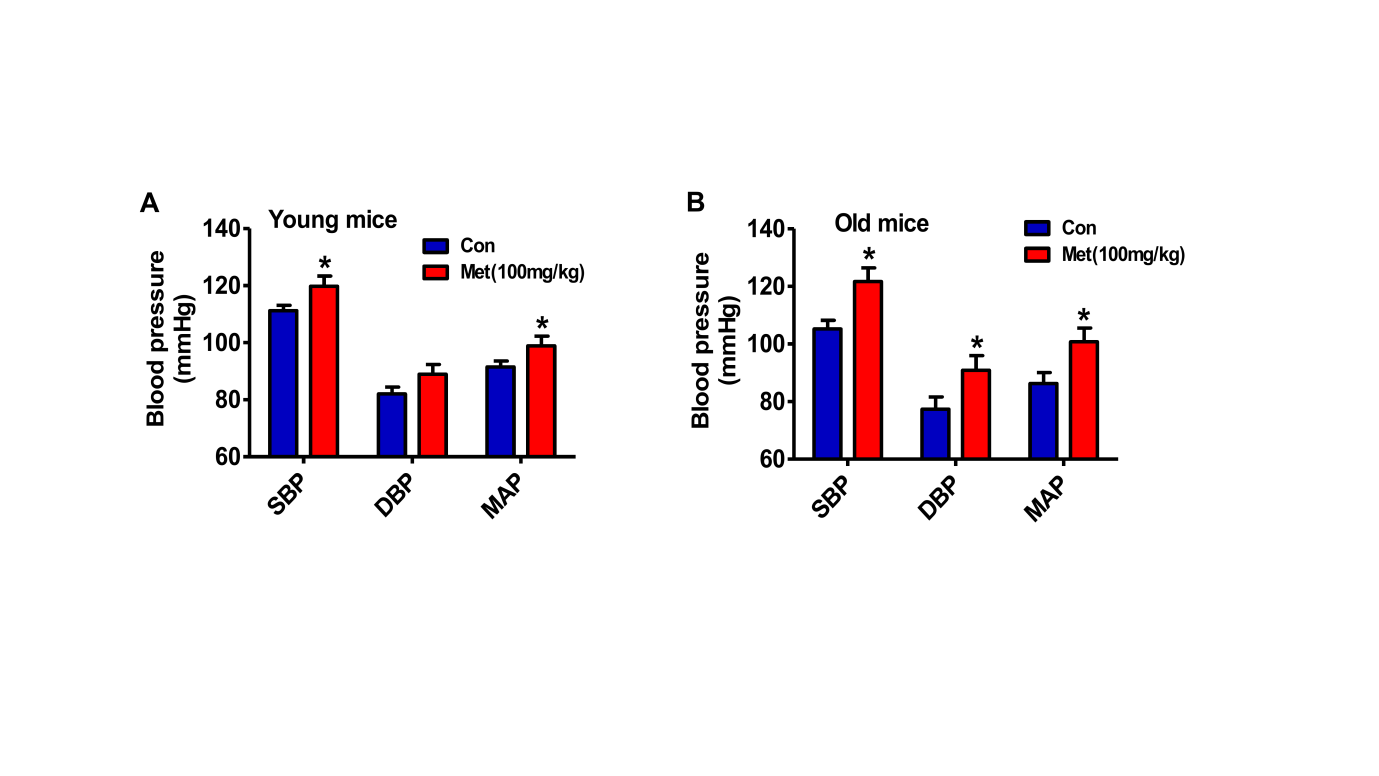


**Supplementary Figure S9. Low dose of metformin on blood pressure levels of young and male C57BL/C mice**. 8-10 week and 60-62 week old male normal C57BL/C mice were orally administrated with metformin (100 mg/kg body weight) for 3 months, respectively, as described in experimental procedure. (A) Blood pressure levels of young mice after 3-month metformin treatment. (B) Blood pressure levels of old mice after 3-month metformin treatment. The blood pressure levels were measured by tail-cuff method. SBP, systolic blood pressure; MAP, mean artery pressure; DBP, diastolic blood pressure. N=8, The results are presented as the mean±SEM. Statistical significance of differences between groups was analyzed by t-test. *P<0.05 versus control group of mice.

**3. Supplementary Tables**

**Table S1. Significant correlations of metformin signature with curated signatures across 10 tissues.**

| **Tissue** | **ID** | **Title of signature** | **GEO** | **Shared Gene** | **SCC** | **Corrected P-value** | **Classification of signature** |
| --- | --- | --- | --- | --- | --- | --- | --- |
| Aorta | case395 | High fat diet-induced atherosclerotic plaque | GSE55796 | 15273 | 0.282 | 3.49E-275 | Deleterious, disease, target organ |
| Aorta | case717 | Atherosclerosis, APOE-deficient model, rescued by captopril treatment | GSE19286 | 16608 | 0.259 | 2.42E-251 | Beneficial, chemical treatment |
| Aorta | case340 | Abdominal aortic aneurysm, porcine pancreatic elastase model | GSE51227 | 15858 | 0.180 | 9.85E-115 | Deleterious, disease, target organ |
| Aorta | case409 | High phosphate diet | GSE57818 | 11309 | 0.211 | 3.58E-113 | Deleterious, lifestyle alteration |
| Aorta | case5 | Abdominal aortic aneurysm, angiotensin II induced | GSE17901 | 16347 | 0.099 | 2.13E-35 | Deleterious, disease, target organ |
| Aorta | case593 | Type 2 diabetes, db/db model | GSE6733 | 12476 | 0.084 | 5.67E-20 | Deleterious, disease, metabolic |
| Aorta | case628 | Diabetes, streptozotocin treatment induced | GSE79623 | 16608 | 0.069 | 9.57E-18 | Deleterious, disease, metabolic |
| Aorta | case716 | Atherosclerosis, APOE-deficient model | GSE19286 | 16608 | 0.057 | 3.06E-12 | Deleterious, disease, metabolic |
| Aorta | case476 | Atherosclerosis, high fat induced (LDLR-/- background) | GSE76812 | 11309 | 0.060 | 1.86E-09 | Deleterious, disease, target organ |
| Aorta | case596 | Atherosclerosis, Ldlr-/- high fat diet model (young) | GSE69187 | 13995 | 0.038 | 7.84E-05 | Deleterious, disease, target organ |
| Aorta | case594 | Aging | GSE69187 | 13995 | 0.028 | 1.60E-02 | Deleterious, other factor |
| Brain | case648 | Parkinson disease, Ercc1-/+ model | GSE75000 | 14827 | -0.160 | 7.64E-84 | Deleterious, disease, target organ |
| Brain | case412 | Angiotensin II treatment | GSE59437 | 16239 | 0.152 | 7.94E-82 | Non-specified, chemical treatment |
| Brain | case178 | Sleep deprivation | GSE9442 | 16239 | -0.148 | 1.36E-77 | Deleterious, lifestyle alteration |
| Brain | case670 | Autism spectrum disorder, Shank2 e6-7 KO model | GSE79824 | 16404 | -0.142 | 2.67E-72 | Deleterious, disease, target organ |
| Brain | case213 | Plasmodium falciparum infection | GSE6019 | 12052 | 0.156 | 3.72E-64 | Deleterious, disease, infectious |
| Brain | case755 | Alzheimer disease (APPswe/PS1dE9 model), rescued by ibuprofen treatment | GSE67306 | 16960 | 0.109 | 1.85E-43 | Beneficial, chemical treatment |
| Brain | case551 | Parkinson's disease, paraquat treatment induced (ventral midbrain) | GSE36232 | 12001 | -0.121 | 3.39E-38 | Deleterious, disease, target organ |
| Brain | case787 | Chronic epilepsy, pilocarpine-induced | GSE77578 | 10292 | 0.122 | 4.50E-33 | Deleterious, disease, target organ |
| Brain | case671 | Autism spectrum disorder, Shank2 e7 KO model | GSE79824 | 16345 | 0.096 | 1.03E-32 | Deleterious, disease, target organ |
| Brain | case187 | Prion disease, ME7 model (hippocampus) | GSE23182 | 16239 | 0.091 | 3.33E-29 | Deleterious, disease, other |
| Brain | case581 | Autism spectrum disorders, BTBR model | GSE81502 | 9949 | -0.115 | 1.88E-28 | Deleterious, disease, target organ |
| Brain | case801 | Depression, unpredictable chronic mild stress induced (cingulate gyrus) | GSE84183 | 15491 | 0.088 | 6.91E-26 | Deleterious, disease, target organ |
| Brain | case20 | Morphine abuse (striatum) | GSE15774 | 12799 | -0.094 | 2.81E-24 | Deleterious, chemical treatment |
| Brain | case638 | Early spinal muscular atrophy, Smn-/- model | GSE102204 | 16776 | -0.078 | 1.53E-21 | Deleterious, disease, other |
| Brain | case25 | Brain ischemia, rescued by DIDS (hippocampus) | GSE12426 | 10129 | 0.096 | 5.40E-20 | Beneficial, chemical treatment |
| Brain | case128 | Huntington disease, YAC128 model | GSE18551 | 16239 | -0.069 | 1.37E-16 | Deleterious, disease, genetic |
| Brain | case654 | Smith-Magenis syndrome, Rai1-/- model (striatum) | GSE81206 | 15272 | 0.068 | 6.74E-15 | Deleterious, disease, genetic |
| Brain | case802 | Unpredictable chronic mild stress induced depression, rescued by fluoxetine treatment (Cingulate Gyrus) | GSE84183 | 15491 | 0.067 | 1.51E-14 | Beneficial, chemical treatment |
| Brain | case117 | Hypoxia challenge (striatum) | GSE19709 | 16239 | 0.064 | 6.43E-14 | Deleterious, other factor |
| Brain | case683 | Sepsis, Lcn2-/-, LPS treatment model | GSE88959 | 12190 | 0.074 | 7.05E-14 | Deleterious, disease, infectious |
| Brain | case123 | 3xTG-AD model of Alzheimer's disease, rescued by pioglitazone | GSE32536 | 16239 | 0.064 | 7.22E-14 | Beneficial, chemical treatment |
| Brain | case505 | Blast traumatic brain injury (hippocampus) | GSE44625 | 10292 | 0.080 | 7.38E-14 | Deleterious, disease, other |
| Brain | case851 | Group 3 medulloblastoma, MYC amplified model | GSE84462 | 15197 | 0.065 | 1.53E-13 | Deleterious, disease, cancer |
| Brain | case661 | Subchronic variable stress challenge (resistant) | GSE85136 | 16296 | 0.063 | 2.11E-13 | Deleterious, other factor |
| Brain | case639 | Late spinal muscular atrophy, Smn-/- model | GSE102204 | 16592 | 0.062 | 2.55E-13 | Deleterious, disease, other |
| Brain | case719 | Huntington disease, HdhQ111 model (striatum) | GSE19780 | 16239 | 0.061 | 9.28E-13 | Deleterious, disease, genetic |
| Brain | case700 | Learning and memory deficit, LSD1 knock-in model | GSE94018 | 16311 | 0.060 | 2.06E-12 | Deleterious, disease, target organ |
| Brain | case722 | Prenatal stress challenge | GSE26025 | 16239 | -0.060 | 2.47E-12 | Deleterious, other factor |
| Brain | case769 | Blast traumatic brain injury, rescued by Exendin-4 treatment | GSE71846 | 10292 | 0.075 | 4.89E-12 | Beneficial, chemical treatment |
| Brain | case329 | Down syndrome, Ts1Cje model (hippocampus) | GSE49635 | 16313 | 0.059 | 1.25E-11 | Deleterious, disease, genetic |
| Brain | case800 | Diet-induced obesity, rescued by Withaferin A treatment | GSE84156 | 16960 | 0.057 | 1.49E-11 | Beneficial, chemical treatment |
| Brain | case410 | Traumatic brain injury | GSE58484 | 16239 | -0.057 | 5.11E-11 | Deleterious, disease, other |
| Brain | case16 | Nicotine abuse (striatum) | GSE15774 | 12799 | -0.063 | 2.40E-10 | Deleterious, chemical treatment |
| Brain | case15 | Heroin abuse (striatum) | GSE15774 | 12799 | -0.061 | 6.33E-10 | Deleterious, chemical treatment |
| Brain | case137 | Quetiapine treatment (frontal cortex) | GSE45229 | 16239 | 0.054 | 7.41E-10 | Non-specified, chemical treatment |
| Brain | case161 | SSH medulloblastoma, Ptch1+/- model | GSE11859 | 16239 | 0.054 | 1.39E-09 | Deleterious, disease, cancer |
| Brain | case753 | Seizure, nicotine-induced (susceptible) | GSE6614 | 8244 | -0.075 | 1.43E-09 | Deleterious, disease, target organ |
| Brain | case19 | Cocaine abuse (striatum) | GSE15774 | 12799 | -0.060 | 1.66E-09 | Deleterious, chemical treatment |
| Brain | case229 | Acute mental stress (water exposure) challenge | GSE40393 | 16960 | -0.052 | 2.86E-09 | Deleterious, lifestyle alteration |
| Brain | case803 | Depression, unpredictable chronic mild stress induced (dentate gyrus) | GSE84183 | 15491 | 0.054 | 4.47E-09 | Deleterious, disease, target organ |
| Brain | case163 | Resveratrol treatment | GSE11291 | 16239 | -0.052 | 5.12E-09 | Beneficial, chemical treatment |
| Brain | case577 | Huntington's disease, N171-HD82Q model (hippocampus) | GSE44855 | 16960 | 0.051 | 5.41E-09 | Deleterious, disease, genetic |
| Brain | case170 | Parkinson disease, 1-methyl-4-phenyl-1,2,3,6-tetrahydropyridine induced (frontal cortex) | GSE7707 | 16239 | -0.051 | 9.04E-09 | Deleterious, disease, target organ |
| Brain | case728 | LPS challenge (young mice) | GSE3253 | 16239 | 0.051 | 9.76E-09 | Deleterious, chemical treatment |
| Brain | case402 | Rett syndrome, MeCP2-/- model | GSE56780 | 16239 | -0.051 | 2.07E-08 | Deleterious, disease, genetic |
| Brain | case777 | Short-term calorie restriction | GSE75569 | 16960 | 0.049 | 3.99E-08 | Beneficial, lifestyle alteration |
| Brain | case350 | Autism spectrum disorder, En2-/- model (Cerebellum) | GSE51612 | 8495 | -0.068 | 4.68E-08 | Deleterious, disease, target organ |
| Brain | case718 | Huntington disease, HdhQ111 model (cerebellum) | GSE19780 | 16239 | 0.049 | 7.27E-08 | Deleterious, disease, genetic |
| Brain | case173 | Impaired spatial memory, aging induced (hippocampus) | GSE13799 | 16239 | 0.049 | 9.02E-08 | Deleterious, disease, other |
| Brain | case591 | Obesity, ob/ob model (hypothalamus) | GSE62013 | 12190 | 0.054 | 4.77E-07 | Deleterious, disease, metabolic |
| Brain | case119 | Hypoxia challenge (hippocampus) | GSE19709 | 16239 | 0.044 | 2.96E-06 | Deleterious, other factor |
| Brain | case158 | Alzheimer disease, Tg2576 model (hippocampus) | GSE36237 | 16239 | 0.044 | 3.13E-06 | Deleterious, disease, target organ |
| Brain | case592 | Diabetes, streptozotocin treatment induced (hypothalamus) | GSE62013 | 12190 | 0.050 | 5.91E-06 | Deleterious, disease, metabolic |
| Brain | case219 | DiGeorge syndrome, Df16+/- model | GSE45935 | 16733 | 0.042 | 8.37E-06 | Deleterious, disease, genetic |
| Brain | case54 | Clozapine treatment (striatum) | GSE48954 | 13690 | 0.046 | 9.80E-06 | Non-specified, chemical treatment |
| Brain | case368 | Visual input deprivation | GSE53136 | 9949 | 0.054 | 1.02E-05 | Deleterious, other factor |
| Brain | case174 | APP model of Alzheimer disease, rescued by brain-derived neurotrophic factor treatment | GSE14499 | 16239 | -0.042 | 1.18E-05 | Beneficial, chemical treatment |
| Brain | case227 | Hypertension (hypothalamus) | GSE25076 | 16960 | 0.040 | 4.25E-05 | Deleterious, disease, other |
| Brain | case483 | Autism spectrum disorders, BTBR T+ Itpr3tf/J model (hippocampus) | GSE81501 | 9949 | 0.051 | 6.83E-05 | Deleterious, disease, target organ |
| Brain | case829 | Kabuki syndrome, Kmt2d+/Bgeo model | GSE90836 | 16960 | -0.039 | 8.70E-05 | Deleterious, disease, genetic |
| Brain | case732 | Fetal alcohol syndrome | GSE34305 | 16960 | 0.038 | 0.000113143 | Deleterious, disease, other |
| Brain | case141 | Vitamin-E treatment | GSE8150 | 16239 | -0.039 | 0.000155176 | Beneficial, chemical treatment |
| Brain | case139 | Huntington disease, RB/1 model (brain hemisphere) | GSE3621 | 16239 | 0.038 | 0.000196495 | Deleterious, disease, genetic |
| Brain | case571 | X-linked infantile spasm syndrome, Arx-/Y model | GSE12609 | 16239 | 0.038 | 0.000288901 | Deleterious, disease, genetic |
| Brain | case120 | Hypoxia challenge (cerebral cortex) | GSE19709 | 16239 | 0.037 | 4.68E-04 | Deleterious, other factor |
| Brain | case694 | Reversal learning impairment, maternal high-dose folic acid induced | GSE80587 | 16665 | -0.035 | 0.000807033 | Beneficial, chemical treatment |
| Brain | case608 | Physical exercise (hippocampus) | GSE29075 | 13690 | 0.039 | 0.000890018 | Beneficial, lifestyle alteration |
| Brain | case580 | Alzheimer disease, APPswe/PS1dE9 model | GSE74615 | 15253 | 0.036 | 0.001236692 | Deleterious, disease, target organ |
| Brain | case747 | Long-term intermittent hypoxia | GSE62385 | 16239 | -0.034 | 0.002917483 | Deleterious, other factor |
| Brain | case351 | Autism spectrum disorder, En2-/- model (Hippocampus) | GSE51612 | 8495 | 0.046 | 0.004016097 | Deleterious, disease, target organ |
| Brain | case479 | West Nile virus infection | GSE77192 | 15674 | 0.034 | 0.004321576 | Deleterious, disease, infectious |
| Brain | case273 | Mucopolysaccharidosis VII, Gusb-/- model (hippocampus) | GSE34071 | 12190 | 0.038 | 0.004842756 | Deleterious, disease, genetic |
| Brain | case707 | Seizure, pilocarpine treatment induced | GSE100202 | 16960 | -0.030 | 0.012501466 | Deleterious, disease, target organ |
| Brain | case705 | Acute stress immobilization (amygdala) | GSE100084 | 13690 | 0.034 | 0.012974397 | Deleterious, other factor |
| Brain | case731 | Wolfram syndrome, Wfs1-/- model | GSE33372 | 16960 | -0.030 | 0.014184537 | Deleterious, disease, genetic |
| Brain | case468 | Memantine treatment (hippocampus) | GSE73798 | 13690 | -0.033 | 0.017988259 | Non-specified, chemical treatment |
| Brain | case653 | Smith-Magenis syndrome, Rai1-/- model (cortex) | GSE81206 | 15640 | 0.031 | 0.018925091 | Deleterious, disease, genetic |
| Brain | case191 | Post-traumatic stress disorder, social defeat model | GSE8870 | 7220 | 0.045 | 0.026052926 | Deleterious, disease, target organ |
| Brain | case752 | Seizure, nicotine-induced (resistant) | GSE6614 | 8244 | 0.041 | 0.02907371 | Deleterious, disease, target organ |
| Brain | case318 | Chitosan treatment | GSE33565 | 12716 | 0.033 | 0.040083035 | Beneficial, chemical treatment |
| Brain | case520 | Alzheimer disease, AD11 anti-NGF model (hippocampus) | GSE63617 | 6066 | 0.047 | 0.045498885 | Deleterious, disease, target organ |
| Brown_adipose | case663 | White adipose browning, CL316243 treatment induced | GSE86338 | 14848 | 0.141 | 7.05E-65 | Beneficial, chemical treatment |
| Brown_adipose | case780 | Short-term calorie restriction | GSE75573 | 16000 | 0.112 | 3.39E-44 | Beneficial, lifestyle alteration |
| Brown_adipose | case640 | High fat diet induced obesity, rescued by narciclasine treatment | GSE63268 | 13876 | 0.114 | 4.42E-40 | Beneficial, chemical treatment |
| Brown_adipose | case130 | Type 1 diabetes, NOD model | GSE33891 | 15470 | -0.104 | 1.02E-36 | Deleterious, disease, metabolic |
| Brown_adipose | case644 | High fat diet induced obesity. resistant vs prone | GSE74804 | 15831 | 0.100 | 1.07E-34 | Beneficial, other factor |
| Brown_adipose | case629 | Aging | GSE85718 | 9778 | 0.122 | 5.62E-32 | Deleterious, other factor |
| Brown_adipose | case646 | High fat diet induced obesity, rescued by green tea | GSE71586 | 15658 | -0.076 | 8.31E-20 | Beneficial, chemical treatment |
| Brown_adipose | case353 | Type 2 diabetes, MKR model | GSE51866 | 14967 | -0.075 | 9.56E-19 | Deleterious, disease, metabolic |
| Brown_adipose | case454 | Caloric restriction | GSE70857 | 9778 | 0.092 | 4.16E-18 | Beneficial, lifestyle alteration |
| Brown_adipose | case482 | White adipose tissue browning, R6/2 model (Huntington's disease model) | GSE79711 | 10384 | -0.089 | 4.34E-18 | Deleterious, disease, genetic |
| Brown_adipose | case845 | Liposarcoma, Atgl-/- Hsl-/- model | GSE97910 | 9778 | -0.089 | 3.70E-17 | Deleterious, disease, cancer |
| Brown_adipose | case426 | Insulin resistance, cytokine tumor necrosis factor-a treatment induced | GSE62635 | 15470 | -0.069 | 2.96E-16 | Deleterious, disease, metabolic |
| Brown_adipose | case448 | High fat diet (brown adipose tissue) | GSE69608 | 15990 | -0.067 | 5.99E-16 | Deleterious, lifestyle alteration |
| Brown_adipose | case455 | Physical exercise | GSE70857 | 9778 | 0.079 | 1.64E-13 | Beneficial, lifestyle alteration |
| Brown_adipose | case10 | Reduced body adiposity, beta-carotene treatment induced | GSE27271 | 15197 | -0.061 | 2.16E-12 | Beneficial, chemical treatment |
| Brown_adipose | case643 | Adipocyte hypertrophy, Nck2-/- model | GSE63510 | 15162 | -0.060 | 4.18E-12 | Deleterious, disease, target organ |
| Brown_adipose | case363 | Pancreatic cancer-induced cachexia syndrome | GSE51931 | 16000 | 0.056 | 5.68E-11 | Deleterious, disease, metabolic |
| Brown_adipose | case405 | High fat diet induced prediabetes, rescued by pioglitazone treatment | GSE57659 | 8627 | 0.071 | 2.24E-09 | Beneficial, chemical treatment |
| Brown_adipose | case335 | Recurrent Aggregatibacter actinomycetemcomitans infection | GSE50647 | 12989 | 0.047 | 2.68E-06 | Deleterious, disease, infectious |
| Brown_adipose | case404 | High fat diet induced prediabetes, rescued by life-style intervention | GSE57659 | 8627 | 0.054 | 2.13E-05 | Beneficial, lifestyle alteration |
| Brown_adipose | case523 | Cold acclimation | GSE13432 | 15470 | 0.038 | 1.04E-04 | Beneficial, other factor |
| Brown_adipose | case85 | Lipodystrophy, Srebf1 mutant model | GSE9130 | 15470 | 0.035 | 4.80E-04 | Deleterious, disease, target organ |
| Brown_adipose | case267 | Rosiglitazone treatment | GSE14810 | 11800 | -0.035 | 7.32E-03 | Beneficial, chemical treatment |
| Brown_adipose | case397 | White adipose tissue-beige transition blockage, CL316243 treatment induced | GSE55934 | 10384 | 0.034 | 1.87E-02 | Deleterious, chemical treatment |
| Brown_adipose | case698 | White adipose tissue browning, Egr1 -/- model | GSE91058 | 16036 | -0.026 | 4.22E-02 | Beneficial, other factor |
| Eye | case330 | Dexamethasone treatment | GSE49872 | 16577 | -0.280 | 2.08E-294 | Beneficial, chemical treatment |
| Eye | case567 | Autosomal recessive retinitis pigmentosa, C2Orf71-/- model | GSE63810 | 15270 | -0.210 | 2.01E-150 | Deleterious, disease, genetic |
| Eye | case377 | Retinal damage, chir99021 induced | GSE54056 | 16577 | -0.133 | 4.94E-65 | Deleterious, disease, target organ |
| Eye | case589 | Diabetic retinopathy, db/db model | GSE55389 | 17389 | -0.109 | 1.07E-45 | Deleterious, disease, metabolic |
| Eye | case317 | Retinal degeneration slow mouse | GSE33134 | 16577 | -0.108 | 3.77E-43 | Deleterious, disease, target organ |
| Eye | case529 | Retinitis pigmentosa, Rhod-/- model | GSE35386 | 16577 | 0.094 | 3.24E-32 | Deleterious, disease, genetic |
| Eye | case556 | Axonal injury, optic nerve crush induced | GSE55228 | 14759 | -0.098 | 2.11E-31 | Deleterious, disease, target organ |
| Eye | case649 | Retinal degeneration, Abca4-/- Rdh8-/- model | GSE75470 | 15350 | -0.095 | 6.71E-31 | Deleterious, disease, target organ |
| Eye | case568 | CRX-associated retinopathies, E168D2 model | GSE65506 | 11174 | -0.102 | 5.91E-26 | Deleterious, disease, target organ |
| Eye | case530 | Retinitis pigmentosa, rd1 model | GSE35386 | 16577 | 0.081 | 2.81E-24 | Deleterious, disease, genetic |
| Eye | case723 | Severe glaucoma, DBA/2J model | GSE26299 | 16577 | -0.073 | 1.50E-19 | Deleterious, disease, target organ |
| Eye | case855 | Aging (male) | GSE95220 | 8626 | -0.088 | 8.22E-15 | Deleterious, other factor |
| Eye | case633 | Aging | GSE22317 | 16577 | -0.059 | 9.65E-13 | Deleterious, other factor |
| Eye | case316 | Leber congenital amaurosis, Rpe65-/- model | GSE3249 | 16577 | 0.056 | 9.79E-12 | Deleterious, disease, genetic |
| Eye | case756 | Hypoglycemia, insulin-induced | GSE67523 | 16176 | 0.056 | 1.71E-11 | Deleterious, disease, metabolic |
| Eye | case300 | Relative axial myopia, Egr1-/- model | GSE16974 | 16577 | 0.051 | 9.45E-10 | Deleterious, disease, target organ |
| Eye | case306 | Hyperoxia challenge | GSE23437 | 16577 | 0.044 | 3.31E-07 | Deleterious, other factor |
| Eye | case308 | Cataracts, Tdrd7-/- model | GSE25776 | 14019 | -0.038 | 1.58E-04 | Deleterious, disease, target organ |
| Eye | case714 | Diabetic retinopathy, streptozotocin treatment induced | GSE12610 | 16577 | 0.031 | 1.29E-03 | Deleterious, disease, metabolic |
| Eye | case522 | Age-related macular degeneration, 5XFAD model | GSE85408 | 11342 | -0.035 | 3.75E-03 | Deleterious, disease, target organ |
| Eye | case854 | Aging (female) | GSE95220 | 8637 | 0.036 | 2.03E-02 | Deleterious, other factor |
| Heart | case846 | Obesity, high fat diet induced | GSE98226 | 15847 | -0.142 | 1.34E-70 | Deleterious, disease, metabolic |
| Heart | case779 | Short-term calorie restriction | GSE75572 | 16028 | 0.134 | 8.01E-64 | Beneficial, lifestyle alteration |
| Heart | case499 | Ovarectomization challenge | GSE34807 | 15522 | 0.103 | 4.27E-36 | Deleterious, other factor |
| Heart | case501 | Ovarectomization challenge, rescued by 17beta-estradiol treatment | GSE34807 | 15522 | -0.082 | 9.60E-23 | Beneficial, chemical treatment |
| Heart | case733 | Chronic myocarditis, coxsackievirus B3 infection induced | GSE35182 | 16028 | -0.070 | 5.99E-17 | Deleterious, disease, infectious |
| Heart | case597 | Aging | GSE72888 | 13713 | 0.074 | 1.55E-16 | Deleterious, other factor |
| Heart | case42 | Doxorubicin challenge | GSE33626 | 13026 | -0.073 | 4.83E-15 | Deleterious, chemical treatment |
| Heart | case401 | Physiological cardiac hypertrophy, voluntary wheel training induced | GSE56348 | 16028 | 0.064 | 3.70E-14 | Deleterious, lifestyle alteration |
| Heart | case430 | Doxorubicin induced cardiotoxicity, rescued by folic acid | GSE64476 | 16028 | -0.063 | 6.61E-14 | Beneficial, chemical treatment |
| Heart | case691 | Eccentric hypertrophy, Cdk8 overexpression model | GSE97027 | 15412 | -0.057 | 6.23E-11 | Deleterious, disease, target organ |
| Heart | case153 | pan-PPAR agonist PPM-201 treatment | GSE31561 | 15522 | -0.057 | 8.29E-11 | Non-specified, chemical treatment |
| Heart | case748 | Cachexia syndrome, C26 model | GSE63032 | 16028 | -0.054 | 5.62E-10 | Deleterious, disease, metabolic |
| Heart | case669 | Maternal caffeine exposure | GSE79013 | 14716 | -0.053 | 8.58E-09 | Deleterious, other factor |
| Heart | case33 | Dilated cardiomyopathy, Ptger4-/- model | GSE16909 | 13026 | -0.054 | 4.19E-08 | Deleterious, disease, target organ |
| Heart | case738 | Diabetes, streptozotocin treatment induced | GSE4616 | 8005 | 0.068 | 5.62E-08 | Deleterious, disease, metabolic |
| Heart | case539 | Cardiac hypertrophy, angiotensin II induced | GSE47420 | 13026 | -0.052 | 2.29E-07 | Deleterious, disease, target organ |
| Heart | case739 | Familial hypertrophic cardiomyopathy (severe vs mild) | GSE4678 | 11794 | 0.053 | 5.59E-07 | Deleterious, disease, genetic |
| Heart | case446 | Dilated cardiomyopathy, PKCe model | GSE68857 | 11794 | 0.053 | 5.68E-07 | Deleterious, disease, target organ |
| Heart | case413 | Angiotensin II treatment | GSE59437 | 15522 | 0.044 | 2.82E-06 | Deleterious, chemical treatment |
| Heart | case475 | Huntington Disease, BACHD model | GSE76593 | 9803 | 0.050 | 5.10E-05 | Deleterious, disease, genetic |
| Heart | case150 | Atorvastatin treatment | GSE23101 | 15522 | 0.039 | 5.15E-05 | Non-specified, chemical treatment |
| Heart | case399 | Cardiac hypertrophy with preserved ventricular function, transverse aortic constriction induced | GSE56348 | 16028 | 0.039 | 5.89E-05 | Deleterious, disease, target organ |
| Heart | case585 | Type 2 diabetes, db/db model | GSE36875 | 15245 | -0.033 | 2.44E-03 | Deleterious, disease, metabolic |
| Heart | case257 | Friedreich's ataxia, KIKO model | GSE15848 | 9085 | 0.040 | 8.32E-03 | Deleterious, disease, genetic |
| Heart | case810 | Cardiac fibrosis, DSC2 overexpression model | GSE84645 | 16028 | -0.029 | 1.28E-02 | Deleterious, disease, target organ |
| Heart | case507 | Post-traumatic stress disorder | GSE52866 | 14669 | -0.028 | 4.05E-02 | Deleterious, disease, target organ |
| Kidney | case697 | Diabetic nephropathy, D2.B6-Ins2Akita/MatbJ | GSE87899 | 15344 | -0.168 | 6.81E-96 | Deleterious, disease, metabolic |
| Kidney | case218 | Renal clear cell adenocarcinoma, VhL mutant model | GSE37464 | 16059 | -0.137 | 2.88E-66 | Deleterious, disease, cancer |
| Kidney | case582 | Diabetic nephropathy, OVE26model | GSE20844 | 15652 | -0.138 | 1.19E-65 | Deleterious, disease, metabolic |
| Kidney | case154 | pan-PPAR agonist PPM-201 treatment | GSE31561 | 15652 | 0.132 | 8.84E-60 | Non-specified, chemical treatment |
| Kidney | case2 | Hydronephrosis, Mgb-/- model | GSE48041 | 15015 | -0.128 | 3.33E-54 | Deleterious, disease, target organ |
| Kidney | case848 | Lupus nephritis, induced by nephrotoxic serum | GSE98626 | 15832 | -0.116 | 6.46E-47 | Deleterious, disease, target organ |
| Kidney | case32 | Fetal alcohol syndrome | GSE23105 | 11715 | -0.133 | 1.84E-45 | Deleterious, disease, other |
| Kidney | case327 | Horse spleen apoferritin induced glomerulonephritis, rescued by captopril | GSE49323 | 11927 | 0.128 | 7.49E-43 | Beneficial, chemical treatment |
| Kidney | case564 | Type 2 diabetes, db/db model | GSE77717 | 14129 | 0.115 | 6.85E-41 | Deleterious, disease, metabolic |
| Kidney | case658 | Acute kidney injury, alcohol and CCl4 treatment induced | GSE83529 | 15349 | -0.110 | 1.29E-40 | Deleterious, disease, target organ |
| Kidney | case735 | Methylmalonic acidemia-associated renal disease, Mut-/- model | GSE41044 | 16237 | 0.102 | 1.94E-37 | Deleterious, disease, genetic |
| Kidney | case604 | Diabetic nephropathy, db/db model | GSE86300 | 14205 | 0.094 | 7.43E-28 | Deleterious, disease, metabolic |
| Kidney | case782 | Renal fibrosis, Escherichia coli induced | GSE76469 | 14850 | -0.089 | 6.59E-26 | Deleterious, disease, target organ |
| Kidney | case659 | Kidney tumor, VhlF/FPbrm1F/FKsp-Cre model | GSE83688 | 8847 | -0.113 | 1.10E-24 | Deleterious, disease, cancer |
| Kidney | case806 | Common bile duct ligation induced cholemic nephropathy, rescued by norursodeoxycholic acid treatment | GSE84584 | 10538 | 0.098 | 1.84E-22 | Beneficial, chemical treatment |
| Kidney | case147 | Glomerulonephritis, Tnfr1-/- model | GSE43928 | 15652 | -0.075 | 2.79E-19 | Deleterious, disease, target organ |
| Kidney | case326 | Glomerulonephritis, horse spleen apoferritin induced | GSE49323 | 11927 | -0.078 | 4.82E-16 | Deleterious, disease, target organ |
| Kidney | case805 | Cholemic nephropathy, common bile duct ligation induced | GSE84584 | 10538 | -0.082 | 1.49E-15 | Deleterious, disease, target organ |
| Kidney | case695 | Fanconi syndrome, conditional Xpr1-/- model | GSE87450 | 15935 | -0.064 | 4.47E-14 | Deleterious, disease, target organ |
| Kidney | case823 | Lupus nephritis, IFNalpha transgenic model | GSE86423 | 14258 | -0.067 | 7.48E-14 | Deleterious, disease, target organ |
| Kidney | case521 | Aging | GSE74463 | 16240 | 0.058 | 5.32E-12 | Deleterious, other factor |
| Kidney | case601 | Candida albicans infection | GSE83680 | 9894 | 0.074 | 6.79E-12 | Deleterious, disease, infectious |
| Kidney | case263 | Doxorubicin challenge | GSE12683 | 11927 | 0.065 | 4.27E-11 | Deleterious, chemical treatment |
| Kidney | case438 | Kidney hyperplasia, vinylidene chloride treatment induced | GSE67320 | 15652 | 0.043 | 2.49E-06 | Deleterious, disease, target organ |
| Kidney | case374 | Uninephrectomy challenge | GSE53996 | 14597 | 0.043 | 1.25E-05 | Deleterious, other factor |
| Kidney | case182 | Hereditary nephrotic syndrome, ICGN model | GSE45005 | 15652 | -0.040 | 2.50E-05 | Deleterious, disease, genetic |
| Kidney | case414 | Angiotensin II treatment | GSE59437 | 15652 | -0.037 | 1.35E-04 | Deleterious, chemical treatment |
| Kidney | case288 | Low dose rate irradiation challenge | GSE14290 | 5153 | 0.065 | 1.40E-04 | Deleterious, other factor |
| Kidney | case376 | Hypertensive kidney | GSE54015 | 16042 | 0.033 | 1.23E-03 | Deleterious, disease, target organ |
| Kidney | case532 | Sepsis, cecal ligation and puncture induced | GSE40180 | 13168 | 0.035 | 2.86E-03 | Deleterious, disease, infectious |
| Kidney | case816 | Diabetic nephropathy, RenTg/Ins2Akita model (susceptible) | GSE85569 | 11927 | 0.037 | 3.07E-03 | Deleterious, disease, metabolic |
| Kidney | case437 | Renal cell carcinoma, vinylidene chloride treatment induced | GSE67320 | 15652 | -0.031 | 5.28E-03 | Deleterious, disease, cancer |
| Liver | case668 | Accelerated aging (Ercc1 delta/- model), rescued by dietary restriction | GSE77494 | 15510 | 0.258 | 2.72E-231 | Beneficial, chemical treatment |
| Liver | case389 | Tumor necrosis factor-alpha treatment | GSE55084 | 11823 | -0.201 | 2.45E-106 | Non-specified, chemical treatment |
| Liver | case435 | Hepatoblastoma, bromodichloroacetic acid treatment induced | GSE67316 | 15598 | -0.152 | 1.32E-78 | Deleterious, disease, cancer |
| Liver | case712 | Non-alcoholic fatty liver disease, rescued by CMPF treatment | GSE106639 | 15598 | -0.139 | 2.51E-66 | Beneficial, chemical treatment |
| Liver | case391 | Beta-naphthoflavone treatment | GSE55084 | 11823 | 0.158 | 4.49E-65 | Non-specified, chemical treatment |
| Liver | case641 | High fat diet induced obesity, rescued by narciclasine treatment | GSE63268 | 13427 | -0.146 | 2.86E-62 | Beneficial, chemical treatment |
| Liver | case387 | Phenylhydrazine treatment | GSE55084 | 11823 | -0.154 | 1.33E-61 | Deleterious, chemical treatment |
| Liver | case221 | Metabolic syndrome X, prenatal undernutrition induced | GSE12117 | 16137 | -0.127 | 9.57E-57 | Deleterious, disease, metabolic |
| Liver | case396 | Cyclosporine A challenge | GSE55881 | 14360 | -0.134 | 3.07E-56 | Deleterious, chemical treatment |
| Liver | case95 | Propylene glycol mono-t-butyl ether challenge | GSE18858 | 15598 | -0.126 | 1.84E-54 | Deleterious, chemical treatment |
| Liver | case762 | Methapyrilene treatment | GSE68364 | 15598 | 0.123 | 7.35E-52 | Non-specified, chemical treatment |
| Liver | case345 | Tetracycline treatment | GSE51543 | 14360 | -0.122 | 7.68E-47 | Deleterious, chemical treatment |
| Liver | case380 | Aroclor 1260 challenge | GSE55084 | 11823 | 0.135 | 8.98E-47 | Deleterious, chemical treatment |
| Liver | case343 | Acetaminophen treatment | GSE51542 | 14360 | -0.118 | 3.67E-43 | Non-specified, chemical treatment |
| Liver | case696 | Hepatoblastoma, beta-catenin and YAP mutation model | GSE87578 | 14161 | -0.118 | 1.13E-42 | Deleterious, disease, cancer |
| Liver | case36 | Schistosoma japonicum infection | GSE25713 | 13051 | -0.120 | 1.41E-40 | Deleterious, disease, infectious |
| Liver | case367 | Obesity induced by high fat diet, rescued by green unroasted coffee | GSE53131 | 15598 | -0.108 | 1.95E-39 | Beneficial, lifestyle alteration |
| Liver | case550 | 70 % partial hepatectomy | GSE78170 | 14754 | 0.108 | 1.12E-37 | Deleterious, other factor |
| Liver | case87 | Prednisolone treatment | GSE21048 | 15598 | -0.105 | 1.35E-37 | Non-specified, chemical treatment |
| Liver | case388 | TCDD challenge | GSE55084 | 11823 | 0.116 | 9.04E-35 | Deleterious, chemical treatment |
| Liver | case93 | 1,4-dichlorobenzene challenge | GSE18858 | 15598 | 0.097 | 5.47E-32 | Deleterious, chemical treatment |
| Liver | case390 | WY-14643 challenge | GSE55084 | 11823 | -0.111 | 1.63E-31 | Deleterious, chemical treatment |
| Liver | case179 | Streptozotocin-induced diabetes, rescued by phlorizin | GSE37415 | 15598 | -0.095 | 1.19E-30 | Beneficial, chemical treatment |
| Liver | case197 | Omega-3 polyunsaturated fatty acids diet | GSE29572 | 15598 | -0.094 | 1.79E-29 | Beneficial, lifestyle alteration |
| Liver | case610 | Aging | GSE36836 | 15598 | 0.093 | 2.02E-29 | Deleterious, other factor |
| Liver | case344 | Amiodarone treatment | GSE51543 | 14360 | 0.096 | 7.15E-29 | Non-specified, chemical treatment |
| Liver | case80 | Dwarfism and longevity, Ames (df/df) model | GSE3150 | 15598 | 0.089 | 9.00E-27 | Beneficial, other factor |
| Liver | case342 | Paraquat challenge | GSE51542 | 14360 | -0.093 | 1.54E-26 | Deleterious, chemical treatment |
| Liver | case489 | Hepatocellular carcinoma, STAM model | GSE83596 | 15895 | -0.085 | 8.96E-25 | Deleterious, disease, cancer |
| Liver | case422 | 1-methoxy-3-indolylmethyl alcohol challenge | GSE61494 | 15598 | 0.079 | 9.84E-21 | Deleterious, chemical treatment |
| Liver | case766 | Sepsis, fecal peritonitis induced | GSE70714 | 9803 | -0.098 | 2.44E-20 | Deleterious, disease, infectious |
| Liver | case436 | Hepatocellular carcinoma, bromodichloroacetic acid treatment induced | GSE67316 | 15598 | -0.078 | 3.89E-20 | Deleterious, disease, cancer |
| Liver | case834 | Sleep deprivation | GSE92913 | 10488 | -0.094 | 4.58E-20 | Deleterious, lifestyle alteration |
| Liver | case194 | Gaucher Disease, Gba1 D409V/null model | GSE23408 | 15598 | -0.077 | 5.17E-20 | Deleterious, disease, genetic |
| Liver | case46 | Type 1 diabetes, rescued by leptin | GSE48598 | 13051 | -0.083 | 1.98E-19 | Beneficial, chemical treatment |
| Liver | case236 | Pirinixic acid (WY-14643) treatment | GSE47844 | 9568 | 0.096 | 4.83E-19 | Deleterious, chemical treatment |
| Liver | case356 | 60% fructose diet | GSE51885 | 15598 | -0.075 | 1.14E-18 | Deleterious, lifestyle alteration |
| Liver | case9 | Post-traumatic stress disorder, social defeat model | GSE58275 | 15332 | 0.075 | 1.22E-18 | Deleterious, disease, target organ |
| Liver | case729 | Hutchinson-Gilford progeria syndrome, LmnaG609G/G609G knock-in model | GSE32609 | 16137 | -0.073 | 1.77E-18 | Deleterious, disease, genetic |
| Liver | case590 | Type 2 diabetes, db/db model | GSE59930 | 15598 | 0.073 | 7.28E-18 | Deleterious, disease, metabolic |
| Liver | case245 | Alcohol induced fatty liver, rescued by garlic oil | GSE40334 | 9803 | 0.090 | 9.93E-17 | Beneficial, chemical treatment |
| Liver | case615 | Overnight fasting (old) | GSE43691 | 9803 | 0.089 | 1.80E-16 | Deleterious, lifestyle alteration |
| Liver | case386 | Phenobarbital challenge | GSE55084 | 11823 | 0.079 | 8.51E-16 | Deleterious, chemical treatment |
| Liver | case252 | Trichloroethylene challenge | GSE24278 | 7772 | 0.098 | 9.00E-16 | Deleterious, chemical treatment |
| Liver | case334 | Aflibercept treatment | GSE50519 | 9675 | -0.087 | 1.60E-15 | Non-specified, chemical treatment |
| Liver | case360 | DHA-supplemented diet | GSE51885 | 15598 | -0.068 | 3.80E-15 | Beneficial, lifestyle alteration |
| Liver | case841 | Diet-Induced nonalcoholic steatohepatitis, rescued by astaxanthin treatment | GSE93819 | 15598 | -0.065 | 6.71E-14 | Beneficial, chemical treatment |
| Liver | case737 | Precancerous liver diseases, Mdr2-/- model | GSE4612 | 11823 | 0.074 | 1.54E-13 | Deleterious, disease, target organ |
| Liver | case279 | Resveratrol treatment | GSE11845 | 10179 | 0.077 | 1.49E-12 | Beneficial, chemical treatment |
| Liver | case96 | Naphthalene challenge | GSE18858 | 15598 | 0.062 | 1.55E-12 | Deleterious, chemical treatment |
| Liver | case142 | C.I Direct Black challenge | GSE44783 | 15598 | 0.061 | 2.57E-12 | Deleterious, chemical treatment |
| Liver | case844 | Non-alcoholic steatohepatitis, choline- and folate-deficient diet induced | GSE96936 | 15332 | -0.061 | 5.18E-12 | Deleterious, disease, target organ |
| Liver | case645 | High fat diet induced obesity. Resistant vs Prone | GSE74804 | 14511 | 0.063 | 7.07E-12 | Beneficial, other factor |
| Liver | case232 | Fasting challenge | GSE46495 | 15945 | 0.059 | 8.58E-12 | Deleterious, lifestyle alteration |
| Liver | case99 | Yersinia pestis infection | GSE18293 | 15598 | -0.060 | 9.12E-12 | Deleterious, disease, infectious |
| Liver | case614 | Overnight fasting (young) | GSE43691 | 9803 | 0.074 | 3.08E-11 | Deleterious, lifestyle alteration |
| Liver | case495 | Fenofibrate treatment | GSE32706 | 15598 | 0.057 | 1.04E-10 | Beneficial, chemical treatment |
| Liver | case533 | Sepsis, cecal ligation and puncture induced | GSE40180 | 13051 | 0.062 | 1.40E-10 | Deleterious, disease, infectious |
| Liver | case494 | Fish oil treatment | GSE32706 | 15598 | 0.055 | 1.17E-09 | Beneficial, chemical treatment |
| Liver | case7 | Hypothyroidism | GSE21307 | 15332 | 0.055 | 1.91E-09 | Deleterious, disease, other |
| Liver | case146 | Piperonyl-butoxide challenge | GSE44783 | 15598 | 0.054 | 3.03E-09 | Deleterious, chemical treatment |
| Liver | case339 | Delayed aging, GHRH-KO model | GSE51108 | 15598 | 0.053 | 6.11E-09 | Beneficial, other factor |
| Liver | case493 | CCl4-induced liver fibrosis, rescued by erlotinib | GSE27640 | 9803 | 0.063 | 4.97E-08 | Beneficial, chemical treatment |
| Liver | case8 | Concanavalin-A induced acute hepatitis, rescued by adipose tissue derived stromal stem cells | GSE41465 | 15332 | -0.051 | 5.29E-08 | Beneficial, other factor |
| Liver | case362 | High fat diet | GSE51885 | 15598 | 0.048 | 2.60E-07 | Deleterious, lifestyle alteration |
| Liver | case542 | Gammaherpesvirus-68 infection | GSE51365 | 15598 | 0.047 | 5.23E-07 | Deleterious, disease, infectious |
| Liver | case612 | Type 2 diabetes, db/db model (young) | GSE43691 | 9803 | 0.059 | 8.16E-07 | Deleterious, disease, metabolic |
| Liver | case94 | 1,2,3-trichloropropane challenge | GSE18858 | 15598 | -0.046 | 1.28E-06 | Deleterious, chemical treatment |
| Liver | case381 | Ciprofibrate treatment | GSE55084 | 11823 | 0.052 | 1.72E-06 | Beneficial, chemical treatment |
| Liver | case525 | Fulminant hepatitis, ConA induced | GSE17184 | 15598 | -0.045 | 2.16E-06 | Deleterious, disease, target organ |
| Liver | case667 | Accelerated aging, Ercc1 delta/- model | GSE77494 | 15511 | 0.045 | 2.52E-06 | Deleterious, disease, other |
| Liver | case793 | Hepatoblastoma, beta-catenin deltaEx3 model | GSE79084 | 14754 | 0.046 | 3.27E-06 | Deleterious, disease, cancer |
| Liver | case606 | High fat diet induced obesity, rescued by methotrexate treatment | GSE87729 | 14409 | 0.046 | 5.42E-06 | Beneficial, chemical treatment |
| Liver | case254 | Oil palm phenolics treatment | GSE28824 | 6499 | -0.062 | 9.66E-05 | Beneficial, chemical treatment |
| Liver | case271 | Retinoic acid treatment | GSE50028 | 11823 | 0.046 | 1.02E-04 | Beneficial, chemical treatment |
| Liver | case602 | Candida albicans infection | GSE83680 | 9803 | 0.050 | 0.000108494 | Deleterious, disease, infectious |
| Liver | case184 | High fat diet | GSE53131 | 15598 | 0.040 | 1.09E-04 | Deleterious, lifestyle alteration |
| Liver | case588 | Obesity, ob/ob model | GSE40775 | 11823 | 0.044 | 0.000232277 | Deleterious, disease, metabolic |
| Liver | case440 | Steatohepatitis, high fat diet induced | GSE67680 | 13051 | 0.042 | 0.000260938 | Deleterious, disease, target organ |
| Liver | case359 | Atherogenic rodent diet | GSE51885 | 15598 | 0.037 | 4.55E-04 | Deleterious, lifestyle alteration |
| Liver | case488 | Liver fibrosis, STAM model | GSE83596 | 15895 | 0.036 | 0.000715346 | Deleterious, disease, target organ |
| Liver | case160 | Hypercholesterolemia, SCD1-/- model | GSE3889 | 15598 | 0.035 | 1.39E-03 | Deleterious, disease, metabolic |
| Liver | case354 | Type 2 diabetes, MKR model | GSE51866 | 15062 | 0.034 | 4.18E-03 | Deleterious, disease, metabolic |
| Liver | case107 | Hypoxia challenge | GSE17796 | 15598 | -0.033 | 5.25E-03 | Deleterious, other factor |
| Liver | case341 | Isoniazid treatment | GSE51542 | 14360 | -0.034 | 6.96E-03 | Deleterious, chemical treatment |
| Liver | case62 | Hepatocellular carcinoma | GSE50431 | 13051 | -0.034 | 0.013814946 | Deleterious, disease, cancer |
| Liver | case487 | Liver non-alcoholic steatohepatitis, STAM model | GSE83596 | 15895 | -0.031 | 0.015291271 | Deleterious, disease, target organ |
| Liver | case730 | Cystic fibrosis, Cftr-/- model | GSE33319 | 9803 | 0.038 | 2.21E-02 | Deleterious, disease, genetic |
| Liver | case113 | Liver injury, hemorrhagic shock induced | GSE26695 | 15598 | -0.030 | 3.29E-02 | Deleterious, disease, target organ |
| Liver | case398 | Maternal high protein diet | GSE56162 | 15598 | 0.029 | 3.99E-02 | Non-specified, other factor |
| Skeletal_muscle | case642 | High fat diet induced obesity, rescued by narciclasine treatment | GSE63268 | 13611 | 0.238 | 2.47E-172 | Beneficial, chemical treatment |
| Skeletal_muscle | case566 | Spinobulbar muscular atrophy, AR113Q-KRKR model | GSE60691 | 13751 | 0.186 | 1.63E-105 | Deleterious, disease, target organ |
| Skeletal_muscle | case126 | Muscle atrophy, tenotomy induced | GSE25908 | 15539 | 0.170 | 1.74E-99 | Deleterious, disease, target organ |
| Skeletal_muscle | case734 | Down syndrome | GSE39159 | 16086 | 0.157 | 3.49E-87 | Deleterious, disease, genetic |
| Skeletal_muscle | case524 | Castration surgery challenge | GSE16486 | 15539 | 0.150 | 1.21E-77 | Deleterious, other factor |
| Skeletal_muscle | case427 | Dysferlinopathy, Dysf-/- F66 model | GSE62945 | 14551 | 0.154 | 3.13E-76 | Deleterious, disease, genetic |
| Skeletal_muscle | case185 | Congenital muscular dystrophy type 1A, Lama2-/- model | GSE12049 | 15539 | 0.147 | 1.77E-74 | Deleterious, disease, genetic |
| Skeletal_muscle | case234 | Fasting challenge | GSE46495 | 15902 | 0.131 | 5.13E-60 | Deleterious, lifestyle alteration |
| Skeletal_muscle | case125 | Muscle atrophy, hindlimb casting induced | GSE25908 | 15539 | 0.129 | 1.73E-56 | Deleterious, disease, target organ |
| Skeletal_muscle | case681 | Muscle dystrophy, recused by weekly glucocorticoid steroid treatment | GSE95682 | 12607 | -0.141 | 5.83E-55 | Beneficial, chemical treatment |
| Skeletal_muscle | case266 | Rhabdomyosarcoma, Kras-/- p53-/- model | GSE25098 | 11815 | 0.138 | 1.17E-49 | Deleterious, disease, cancer |
| Skeletal_muscle | case631 | Aging, rescued by nicotinamide mononucleotide treatment | GSE85718 | 9820 | 0.132 | 1.85E-37 | Beneficial, chemical treatment |
| Skeletal_muscle | case188 | Cigarette smoke | GSE18033 | 15531 | 0.103 | 6.37E-36 | Deleterious, lifestyle alteration |
| Skeletal_muscle | case442 | Lethal hypoglycemia, Sirt6-/- model | GSE67780 | 15539 | 0.092 | 5.45E-29 | Deleterious, disease, metabolic |
| Skeletal_muscle | case105 | Hyperlipidemia, TALLYHO x C57BL6 F2 model | GSE24637 | 15539 | -0.090 | 8.74E-28 | Deleterious, disease, metabolic |
| Skeletal_muscle | case21 | Muscle atrophy, denervation induced | GSE44205 | 9436 | 0.114 | 8.08E-27 | Deleterious, disease, target organ |
| Skeletal_muscle | case264 | Physical exercise | GSE5297 | 11814 | -0.102 | 8.60E-27 | Beneficial, lifestyle alteration |
| Skeletal_muscle | case156 | pan-PPAR agonist PPM-201 treatment | GSE31561 | 15539 | 0.087 | 6.79E-26 | Non-specified, chemical treatment |
| Skeletal_muscle | case743 | Muscle atrophy, Tp53inp2 overexpression induced | GSE54917 | 14159 | 0.083 | 3.33E-21 | Deleterious, disease, target organ |
| Skeletal_muscle | case622 | Obesity, ob/ob model (old) | GSE43691 | 9820 | 0.098 | 1.35E-20 | Deleterious, disease, metabolic |
| Skeletal_muscle | case678 | Muscular atrophy (Huntington Disease), R6/2 model | GSE81367 | 15903 | 0.076 | 5.10E-20 | Deleterious, disease, genetic |
| Skeletal_muscle | case627 | Pathological aging, Mfn2-/- model | GSE71501 | 14159 | 0.080 | 9.34E-20 | Deleterious, disease, other |
| Skeletal_muscle | case749 | Cachexia syndrome (C26 model) | GSE63032 | 16086 | 0.073 | 1.33E-18 | Deleterious, disease, metabolic |
| Skeletal_muscle | case365 | Pancreatic cancer-induced cachexia syndrome | GSE51931 | 16086 | 0.071 | 1.38E-17 | Deleterious, disease, metabolic |
| Skeletal_muscle | case261 | Skeletal muscle adaption, motor nerve stimulation induced | GSE17620 | 11815 | -0.079 | 6.99E-16 | Non-specified, other factor |
| Skeletal_muscle | case428 | Muscle atrophy, p21 transfection model | GSE63007 | 9820 | 0.076 | 2.11E-12 | Deleterious, disease, target organ |
| Skeletal_muscle | case677 | Hypoxia challenge | GSE81286 | 11617 | -0.070 | 2.45E-12 | Deleterious, other factor |
| Skeletal_muscle | case679 | Muscular atrophy (Huntington Disease), rescued by ActRIIB decoy | GSE81367 | 15956 | -0.059 | 3.31E-12 | Beneficial, chemical treatment |
| Skeletal_muscle | case355 | Type 2 diabetes, MKR model | GSE51866 | 15026 | -0.052 | 1.17E-08 | Deleterious, disease, metabolic |
| Skeletal_muscle | case189 | Myotonic dystrophy | GSE24920 | 15539 | 0.050 | 2.58E-08 | Deleterious, disease, target organ |
| Skeletal_muscle | case456 | Caloric restriction | GSE70857 | 9820 | -0.058 | 4.46E-07 | Beneficial, lifestyle alteration |
| Skeletal_muscle | case512 | Congenital myopathy, cfl2-/- model | GSE61404 | 16086 | -0.045 | 7.75E-07 | Deleterious, disease, genetic |
| Skeletal_muscle | case370 | In utero undernutrition | GSE53520 | 9556 | 0.053 | 1.51E-05 | Deleterious, other factor |
| Skeletal_muscle | case562 | Centronuclear myopathy, Smyd1-/- model | GSE71679 | 14331 | 0.043 | 1.55E-05 | Deleterious, disease, target organ |
| Skeletal_muscle | case473 | Eicosapentaenoic and docosahexaenoic acid enriched diet | GSE76361 | 13056 | -0.044 | 2.24E-05 | Beneficial, lifestyle alteration |
| Skeletal_muscle | case164 | Resveratrol treatment | GSE11291 | 15539 | -0.039 | 6.59E-05 | Beneficial, chemical treatment |
| Skeletal_muscle | case623 | Overnight fasting (old) | GSE43691 | 9820 | 0.048 | 1.04E-04 | Deleterious, lifestyle alteration |
| Skeletal_muscle | case14 | Oculopharyngeal muscular dystrophy, A17.1 model | GSE26604 | 9636 | 0.038 | 9.28E-03 | Deleterious, disease, genetic |
| Skeletal_muscle | case620 | Type 2 diabetes, db/db model (young) | GSE43691 | 9820 | 0.036 | 2.27E-02 | Deleterious, disease, metabolic |
| Skeletal_muscle | case830 | Muscular hypertrophy, conditional Hirs-/- model | GSE90900 | 13421 | 0.030 | 2.99E-02 | Deleterious, disease, target organ |
| Skeletal_muscle | case624 | Type 2 diabetes, db/db model (old) | GSE43691 | 9820 | 0.035 | 3.09E-02 | Deleterious, disease, metabolic |
| Stomach | case666 | Stomach ulcer, acetic acid treatment induced | GSE76565 | 16420 | 0.168 | 3.72E-103 | Deleterious, disease, target organ |
| Stomach | case764 | Obesity, high fat diet induced | GSE69306 | 14938 | -0.082 | 1.19E-22 | Deleterious, disease, metabolic |
| Stomach | case352 | Gastroparesis, dexamethasone treatment induced | GSE51669 | 16069 | -0.077 | 9.26E-22 | Deleterious, disease, target organ |
| Stomach | case74 | Gastric adenocarcinoma, Wnt1/C2mE over expression model | GSE16902 | 16204 | 0.068 | 3.61E-17 | Deleterious, disease, cancer |
| Stomach | case776 | Long-term alcohol treatment | GSE75373 | 9946 | -0.069 | 9.50E-11 | Deleterious, lifestyle alteration |
| Stomach | case857 | Gastritis, Helicobacter pylori infection induced | GSE13873 | 16204 | 0.049 | 3.49E-09 | Deleterious, disease, infectious |
| Stomach | case763 | Obesity, ob/ob model | GSE69306 | 14938 | -0.038 | 4.62E-05 | Deleterious, disease, metabolic |
| Stomach | case459 | Tamoxifen treatment | GSE71580 | 16204 | 0.031 | 1.08E-03 | Deleterious, chemical treatment |
| Stomach | case319 | Chitosan treatment | GSE33565 | 12710 | 0.031 | 5.42E-03 | Beneficial, chemical treatment |
| Stomach | case310 | Lactobacilli inoculation | GSE28895 | 16204 | 0.024 | 3.21E-02 | Beneficial, other factor |
| Testis | case315 | Male infertility, Miwi+/ADH model | GSE32180 | 18048 | 0.119 | 1.74E-56 | Deleterious, disease, target organ |
| Testis | case286 | Heat shock challenge (susceptible) | GSE13010 | 16936 | -0.072 | 9.10E-20 | Deleterious, other factor |
| Testis | case651 | Arrested spermatogenesis, Trf2-/- model | GSE79602 | 16706 | -0.072 | 1.08E-19 | Deleterious, disease, target organ |
| Testis | case322 | Male infertility, Ikbkap -/- model | GSE42230 | 18048 | -0.037 | 1.09E-05 | Deleterious, disease, target organ |
| Testis | case289 | Low dose rate irradiation challenge | GSE14290 | 5399 | -0.058 | 3.46E-04 | Deleterious, other factor |
| Testis | case703 | Male infertility, Mettl3-/- model | GSE99771 | 15618 | -0.031 | 1.81E-03 | Deleterious, disease, target organ |

**Table S2. Predicted drug-drug interactions between the top drugs from network analysis and metformin.**

| **Drug A** | **Drug B** | **Drug-drug interaction description** | **DeepDDI Score** |
| --- | --- | --- | --- |
| Metformin | Carbidopa | The therapeutic efficacy of Metformin can be decreased when used in combination with Carbidopa. | 0.999179 |
| Metformin | Clozapine | The risk or severity of adverse effects can be increased when Metformin is combined with Clozapine. | 0.998207 |
| Metformin | Dyphylline | The serum concentration of Metformin can be increased when it is combined with Dyphylline. | 0.954708 |
| Ketoprofen | Metformin | Ketoprofen may decrease the excretion rate of Metformin which could result in a higher serum level. | 0.603692 |
| Glyburide | Metformin | Glyburide may increase the hypoglycemic activities of Metformin. | 0.93085 |
| Metformin | Rifampicin | The serum concentration of Metformin can be increased when it is combined with Rifampicin. | 0.544103 |
| Rifampicin | Metformin | The serum concentration of Rifampicin can be decreased when it is combined with Metformin. | 0.47254 |
| Tolbutamide | Metformin | Tolbutamide may increase the hypoglycemic activities of Metformin. | 0.993429 |
| Metformin | Rifaximin | The serum concentration of Metformin can be increased when it is combined with Rifaximin. | 0.960725 |
| Gemfibrozil | Metformin | Gemfibrozil may increase the hypoglycemic activities of Metformin. | 0.632285 |
| Metformin | Regadenoson | The risk or severity of adverse effects can be increased when Metformin is combined with Regadenoson. | 0.950113 |
| Metformin | Olanzapine | The therapeutic efficacy of Metformin can be decreased when used in combination with Olanzapine. | 0.999826 |
| Metformin | Clozapine | The therapeutic efficacy of Metformin can be decreased when used in combination with Clozapine. | 0.999821 |
| Metformin | Loxapine | The therapeutic efficacy of Metformin can be decreased when used in combination with Loxapine. | 0.99968 |
| Metformin | Dyphylline | The therapeutic efficacy of Metformin can be decreased when used in combination with Dyphylline. | 0.934906 |
| Metformin | Risperidone | The therapeutic efficacy of Metformin can be decreased when used in combination with Risperidone. | 0.999691 |
| Metformin | Metyrosine | The therapeutic efficacy of Metformin can be decreased when used in combination with Metyrosine. | 0.999385 |
| Pentoxifylline | Metformin | Pentoxifylline may increase the hypotensive activities of Metformin. | 0.957084 |
| Metformin | Ketoprofen | The therapeutic efficacy of Metformin can be decreased when used in combination with Ketoprofen. | 0.990423 |
| Metformin | Glyburide | Metformin may increase the hypoglycemic activities of Glyburide. | 0.999688 |
| Metformin | Tolbutamide | Metformin may increase the hypoglycemic activities of Tolbutamide. | 0.983593 |
| Rifaximin | Metformin | The serum concentration of Rifaximin can be increased when it is combined with Metformin. | 0.934451 |
| Metformin | Quetiapine | The therapeutic efficacy of Metformin can be decreased when used in combination with Quetiapine. | 0.999568 |
| Metformin | Chlorprothixene | The therapeutic efficacy of Metformin can be decreased when used in combination with Chlorprothixene. | 0.999943 |
| Metformin | Midodrine | The therapeutic efficacy of Metformin can be decreased when used in combination with Midodrine. | 0.99987 |
| Metformin | Eletriptan | The therapeutic efficacy of Metformin can be decreased when used in combination with Eletriptan. | 0.998692 |

**Table S3. The altered cell type enrichment score after metformin treatment as estimated by xCell transcriptome deconvolution method.**

| **Tissue** | **Cell type** | **Score (control)** | **Score (metformin)** | **Changes** | **P-value** |
| --- | --- | --- | --- | --- | --- |
| Brown adipose | Hematopoietic stem cell | 0.282 | 0.235 | Down | 1.13E-02 |
| Heart | Natural killer T cell | 0.0588 | 0.0235 | Down | 1.49E-02 |
| Liver | Conventional dendritic cell | 0.0608 | 0.0329 | Down | 3.60E-02 |
| Skeletal muscle | Smooth muscle | 0.140 | 0.175 | Up | 1.57E-02 |
| Skeletal muscle | Adipocyte | 0.0633 | 0.0228 | Down | 2.13E-02 |
